# Skin‐Inspired Design of Self‐Healing Coatings Integrating Interface‐Liquid Repellency and Corrosion Resistance

**DOI:** 10.1002/advs.202521067

**Published:** 2026-02-10

**Authors:** Bingzhi Li, Bingce Liu, Enyu Guo, Zhihao Zhou, Yibo Ouyang, Xiao‐Bo Chen, Huijun Kang, Zongning Chen, Tongmin Wang

**Affiliations:** ^1^ Key Laboratory of Solidification Control and Digital Preparation Technology (Liaoning Province) School of Materials Science and Engineering Dalian University of Technology Dalian P. R. China; ^2^ Ningbo Institute of Dalian University of Technology Ningbo P. R. China; ^3^ Department of Mechanical Manufacturing and Mechatronic Engineering School of Engineering RMIT University Melbourne Victoria Australia; ^4^ Department of Mechanical Engineering Harbin University of Science and Technology Rongcheng P. R. China

**Keywords:** coatings, corrosion, hydrophobic, self‐healing, skin‐inspired

## Abstract

Designing coatings with a wide spectrum of functions such as self‐healing, liquid repellency, anticorrosion, and a high level of mechanical robustness is crucial in engineering applications. However, simultaneously meeting two or more conflicting requirements remains a challenge. In this work, a holistic, skin‐inspired tri‐layer coating is proposed to resolve the conflicting requirements of self‐healing, liquid repellency, and corrosion resistance in hydrophilic polymer materials. The rational design of multiple gradients in self‐healing, wetting, and strength endows a sustained liquid repellency, corrosion resistance, and self‐healing even under harsh environments, as well as strong adhesion with metal substrate. The skin‐inspired tri‐layer coating exhibits complete self‐healing even in harsh aqueous environments, owing to the synergistic interaction between layers. The tri‐layer structure consists of a hydrophobic epidermis‐like barrier layer, a hydrophilic self‐healing polymer middle layer, and a micro‐arc oxidation porous base layer that provide strong interfacial adhesion and mechanical support. The hydrophilic polymer layer, composed of polyvinyl alcohol and tannic acid, rapidly repairs damaged coating regions through hydrogen bonding and diffusion, triggered by water molecules. Meanwhile, the hydrophobic outer layer acts as a sealing barrier, limiting excessive diffusion of the hydrophilic polymer. Such an integrated skin‐inspired coating strategy provides new insights into design and manufacturing multifunctional polymeric coatings to tackle the critical challenges in a variety of engineering services.

## Graphical abstract

A skin‐inspired multilayered coating system is developed to integrate multiple, often conflicting, functions of self‐healing capability, strong liquid repellency, long‐term corrosion resistance, and robust adhesion, to tackle diverse challenges in engineering services. The three‐layer architecture is consisted of a porous bottom layer with strong mechanical adhesion, an intermediate layer with rapid self‐healing; and a top layer with hydrophobicity.

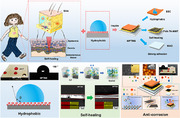

## 1 Introduction

The rapid advancement of materials science, chemical engineering, and nanotechnology has accelerated the development of multifunctional materials [1]. Among these, coatings play as primary interface between substrate and external environment. They offer unique advantages in achieving multifunctionality, including corrosion resistance [2, 3, 4], self‐cleaning [5], and other protective properties [6]. However, conventional coatings remain vulnerable to mechanical damage, and environmental stresses. These factors often lead to surface wear, delamination, and a gradual loss of functionality. Consequently, there is an urgent need to design coating systems with intrinsic self‐healing capabilities to improve durability and extend service lifetimes [7, 8].

To date, two possible strategies have been reported for designing self‐healing coatings [9]. One strategy involves passivating the surface of active materials [10]. Its self‐healing performance primarily depends on the dissolution and re‐precipitation of local defects. Tannic acid (TA) possesses a polyphenol structure with hydroxyl (OH) groups in the ortho position. These groups allow it to react with metal ions, forming TA‐metal complexes [11]. Zhu et al. [12] fabricated a TA‐based composite coating on magnesium (Mg) alloy AZ31 by immersing substrate in an aqueous TA solution. When exposed to water, TA within the coating is rapidly released into the solution, where it chelates with Mg ions generated by corrosion.

The other strategy is to introduce self‐healing polymers with reversible bonds, such as hydrogen bonds, metal‐ligand coordination and ionic interactions, to achieve coating self‐healing [13]. Hydrophilic self‐healing polymers leverage ambient moisture or humidity to activate their self‐repair mechanisms, eliminating the need for external energy or chemical triggers. Wang et al. [14] utilized hydrophilic polymer polyvinyl alcohol (PVA) to provide a micro self‐healing framework for perovskite crystals. Such a framework can heal mechanical damage by absorbing moisture from surrounding environment. Qi et al. [15] mimicked the epidermis‐like structure of the human body by combining hydroxyl groups in PVA with phenolic hydroxyl groups in TA. Through multiple intermolecular hydrogen bonds, they developed a series of self‐healing films.

However, those dynamic bonds are generally unstable in water. Water molecules function as hydrogen bond donors and acceptors, ligands for metal ions, and polar solvents. Consequently, water molecules can interfere with dynamic bonds in materials through multiple mechanisms. By forming competitive hydrogen bonds and coordinating with metal cations, water molecules can weaken existing dynamic bonds, reducing their bond strength. In addition, water absorption and subsequent expansion induce stretching or even fracture of the cross‐linked network, hindering the recombination of dynamic bonds. Ultimately, these effects cause hydrophilic self‐healing materials to jeopardize their self‐healing properties [16]. Therefore, under harsh service conditions, as protective barrier and mediator interacting with external environment, protective coatings must exhibit superior corrosion resistance and strong robustness, and possess durable hydrophobic and self‐healing properties, which remains a challenge to date [17, 18, 19].

Skin provides an effective inspiration for addressing the above challenges [20, 21]. As the largest multifunctional organ in the human body, skin comprises epidermis, dermis, and subcutaneous tissue. It exhibits numerous remarkable characteristics, including effective physical barrier protection, and rapid self‐healing capabilities. As the outermost layer, epidermis primarily consists of the rigid stratum corneum, which serves as a physical barrier and provides waterproof protection. Dermis fulfills critical functions, including dissipating energy from external mechanical deformation, protecting deeper skin tissues, and facilitating rearrangement and repair with fibroblast assistance to promote wound healing. Beneath dermis lies the subcutaneous tissue, composed primarily of loose connective tissue and fat. It acts as a mechanical support base while serving as a strong adhesive interface that tightly connects skin to underlying muscles, enhancing structural cohesion [20, 21]. Such a three‐layer system integrates hydrophobic and rigid barriers (epidermis), rapid healing and recombination (dermis), and strong adhesion and mechanical strength (subcutaneous tissue). Inspired by the hierarchical structure of human skin, Zhang et al. [20, 21]. developed an innovative bioinspired composite through multi‐gradient structural engineering of epoxy resin, silica nanoparticles, and lignin. This layered design approach mirrors the functional integration commonly seen in biological tissues, where distinct layers work together to achieve performance that exceeds the reach of their individual counterparts.

**FIGURE 1:**
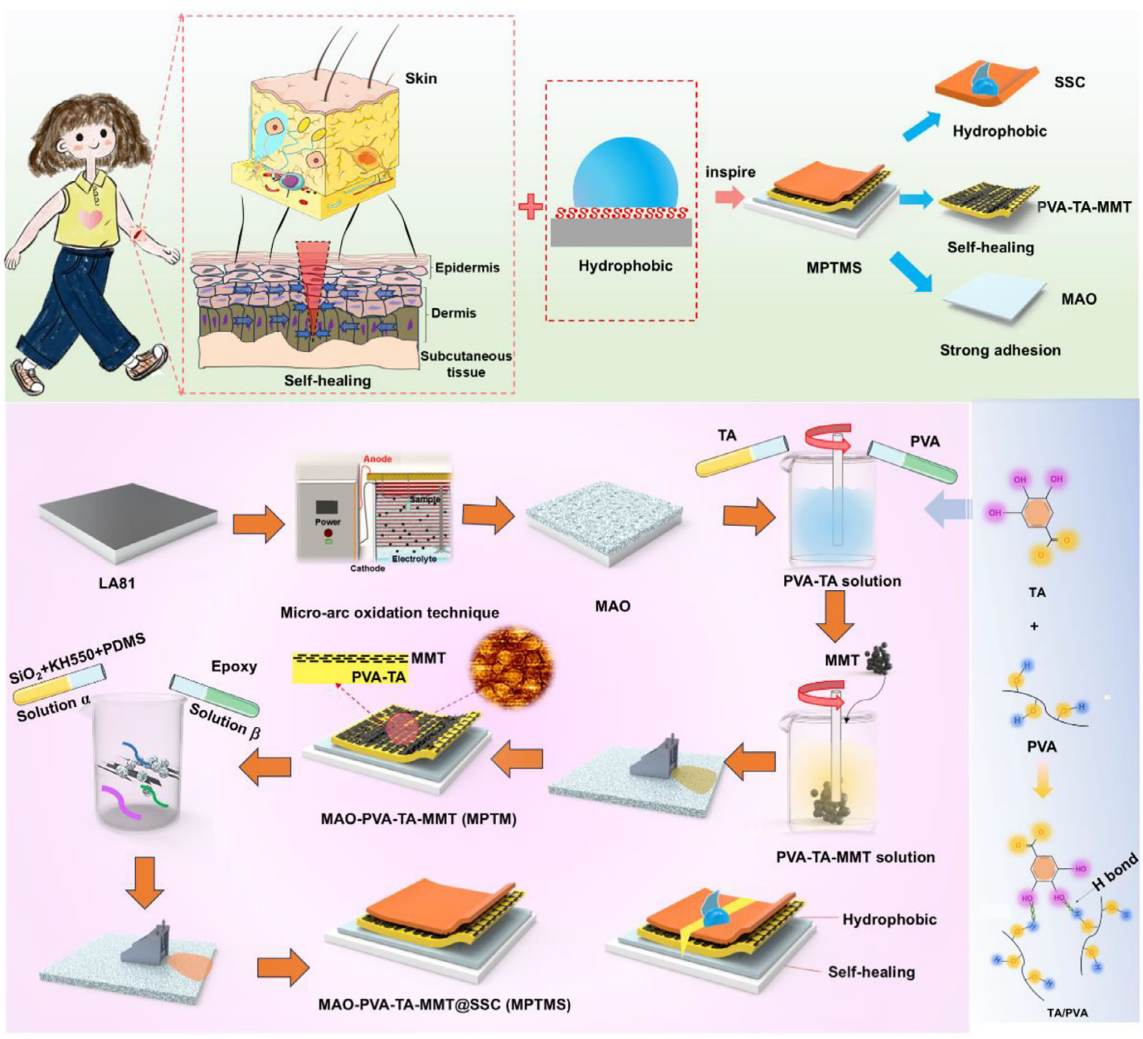
Schematic illustration of the preparation of MPTMS coating upon Mg–Li alloy LA81.

As such, we design and engineer a multilayered coating to replicate the hierarchical structure of human skin to yield hydrophobicity, self‐healing ability, and mechanical strength strategically allocated across individual layers. In brief, the top layer, analogous to epidermis, consists of a solid‐like slippery coating that provides hydrophobicity and acts as a robust physical barrier. The middle layer, analogous to dermis, consists of a hydrophilic self‐healing polymer composed of TA and PVA. Its self‐healing capability is driven by synergistic mechanisms, including metal chelation, molecular diffusion, and dynamic covalent recombination following coating damage. The bottom layer is an in situ grown MAO coating with a porous structure that enhances interfacial adhesion with the middle layer. The self‐healing coating, inspired by the layered structure of skin, effectively addresses the conflicting demands of self‐healing, hydrophobicity, corrosion resistance, and adhesion strength. Such a coating strategy is expected to broaden the engineering applications of hydrophilic self‐healing polymeric coatings (Figure 1).

## 2 Results and Discussion

### 2.1 Coating Fabrication Strategy

TA is a polyphenolic compound that contains multiple phenolic hydroxyl groups (─OH). PVA has a molecular chain rich in hydroxyl groups (─OH). When TA is mixed with PVA, strong hydrogen bonds form between the hydroxyl groups of TA and PVA, binding the two substances together (Figure 1). Partially replacing water with ethanol as the solvent reduces the hydrogen bond density between polymers. This weakens intermolecular interactions and prevents precipitation induced by hydrogen bonding [15]. A homogeneous composite system of TA and PVA is successfully developed through the synergistic effect of solvents. It is achieved by combining thermally induced dissolution with an ethanol‐assisted dispersion strategy. The resulting mixture formed a homogeneous pale lime‐yellow solution with high fluidity (Figure S1a).

Figure S1a demonstrates the characteristic peaks for pure TA, PVA, and the hydrophilic self‐healing polymer PVA‐TA complex (PT). For TA, the peak at 3401 cm^−1^ corresponds to the stretching vibration of the ─OH group, while the peak at 1716 cm^−1^ represents the C═O stretching vibration of ester or carboxylic acid groups. The absorption peak at 1600 cm^−1^ is attributed to the C═C bond in the aromatic ring, and the peak at 1197 cm^−1^ is due to the C─O bond in phenolic and ester groups; while for PVA, the peak at 3342 cm^−1^ is associated with the stretching vibration of the ─OH group, the peak at 2945 cm^−1^ with the stretching vibration of the C─H bond, and the peak at 1092 cm^−1^ with the stretching vibration of the C─O bond. For the PT, the ─OH stretching vibration peak appears at 3296 cm^−1^, the C─H stretching vibration peak at 2942 cm^−1^, and the C═O stretching vibration peak at 1708 cm^−1^. The shift of the ─OH, C─H, and C═O stretching vibration peaks to lower wavenumbers, along with broadening of the band, indicates the formation of hydrogen bonds between TA and PVA molecules [22].

To mimic the structure of skin's stratum corneum, low‐cost montmorillonite with strong physical barrier properties was introduced into PVA‐TA mixed solution. After adding MMT, color of the solution changed from pale lime‐yellow to brown (Figure S1b). FT‐IR spectrum of the hydrophilic self‐healing PVA‐TA‐MMT film (PTM) exhibits a strong Si─O stretching vibration absorption band around 1024 cm^−1^ (Figure S1b). This peak is characteristic of montmorillonite, as reported in the literature. Compared to the typical absorption peak of pure montmorillonite at 1034 cm^−1^, a shift of 10 cm^−1^ is observed. It suggests the formation of specific intermolecular interactions between the polymer matrix and the montmorillonite sheets. The characteristic peaks of MMT and PT appeared simultaneously in the spectrum of the film, indicating that MMT was compatible with PT [23].

X‐ray photoelectron spectroscopy (XPS) analyses were performed to analyze chemical composition, and binding states of the hydrophilic self‐healing MAO‐PVA‐TA‐MMT film (MPTM, Figure S2). XPS survey spectrum confirms the presence of C, O, Si, and Al elements in MPTM. C 1s spectrum of MPTM is deconvoluted to show four peaks located at around 284.7, 286.1, 287.1, and 289.2 eV, which are associated with C─H, C─OH, C─C, and C═O groups, respectively [24, 25]. C─OH/C─O bonds are assigned to PVA, and the C═O is related to TA [26]. O 1s spectrum of MPTM is deconvoluted to show three peaks located at around 531.7, 532.6, and 533.0 eV assigning to C─O, Si─O and C═O groups in PVA molecule [27]. In addition, fine‐scan XPS spectra for Si 2p and Al 2p were deconvoluted into two component peaks. Si 2p signal corresponds to Si─O at 103.5 eV and Al_2_SiO_5_ at 102.8 eV, while Al 2p signal corresponds to Al─O at 74.6 eV and Al_2_SiO_5_ at 74.4 eV. The above results prove that MMT has been introduced into MPTM. MMT sample displays a layered and flaky structure with an average size of 3–6 µm. Flakes are composed primarily of Si, O, Al, and C with minor Na, and Mg dispersed within a matrix surface (Figure S3). Though the addition of a small quantity of MMT micro/nanosheets to PT can slow down the diffusion of the hydrophilic polymer layer in aqueous solutions, however, synthesized MPTM cannot be stabilized well in aqueous solution for a long time due to its high diffusivity in aqueous solution, which limits its applications.

By mimicking hydrophobic model in nature, a hydrophobic layer was constructed on the surface of the hydrophilic polymer. It minimizes the instability of the hydrophilic polymer under high‐humidity conditions. When immersed in ultrapure water for 1 h, the hydrophilic polymer exhibited high diffusivity, causing the solution to shift from colorless and transparent to light yellow (Figure S4). It suggests that aqueous environments are not suitable for preparing composite films that incorporate hydrophilic polymers. The hydrophilic self‐healing polymer, i.e. MPTM, was immersed in a standard ethyl acetate solution. Surface of the coating remained unchanged, with no significant reactions observed after 48 h (Figure S5), indicating that ethyl acetate imposes no adverse effects on the coating. A highly corrosion‐resistant hydrophobic coating (SSC) was synthesized via sequential dehydration, condensation, and cross‐linking reactions in ethyl acetate, employing hydrophilic nano‐silica and commercial reagents, including polydimethylsiloxane (Figures S6‐S8). As shown in Figure S9, the obtained solid‐like slippery solution was homogeneous, opalescent, and highly mobile.

Due to the high flowability of SSC coating, a variety of coating techniques can be employed to fabricate hydrophobic membrane layers. In this study, the doctor blade (DSC) scraping method was used to produce uniform thin films across a large surface area. Shear forces were subsequently applied to form a water‐repellent outer layer that mimics the epidermis of natural skin on MPTM substrate. FTIR spectra of the skin‐like MPTMS coating reveal that vibration peaks at 2965 cm^−1^ are assigned to the stretching vibration band of ─CH_3_ from polydimethylsiloxane. The peak at 1200–1400 cm^−1^ is indicative of Si─CH_3_ stretching vibrations. The broad and intense absorption band at 1093 cm^−1^ is ascribed to the anti‐symmetric stretching vibration peaks of silica nanoparticles, confirming the successful formation of SSC coating containing modified silica nanoparticles (Figure S9) [28, 29]. It was further validated by XPS spectroscopy (Figures S10–S12). No signal of Al from MPTM was detected, indicating that SSC coating is uniform and dense, completely covering the hydrophilic polymer. This hydrophobic layer, serving as the top coating of a biomimetic skin, effectively prevents direct contact between MPTM and aqueous solutions, thereby expanding the application potential of these polymers.

Due to the spark discharge phenomenon during the MAO process of Mg–Li alloys (Figure S13), the surface film formed exhibits a porous structure with varying pore sizes, as shown in Figures S14–S16. This structure functions similarly to the subcutaneous tissue in human skin, mechanically anchoring the alloy substrate to the self‐healing polymer coating. Additionally, unsaturated metal atoms on MMT surface can strongly interact with hydroxyl groups on PVA‐TA film, and SSC film, ensuring firm adhesion [15].

### 2.2 Bonding Strength

**FIGURE 2:**
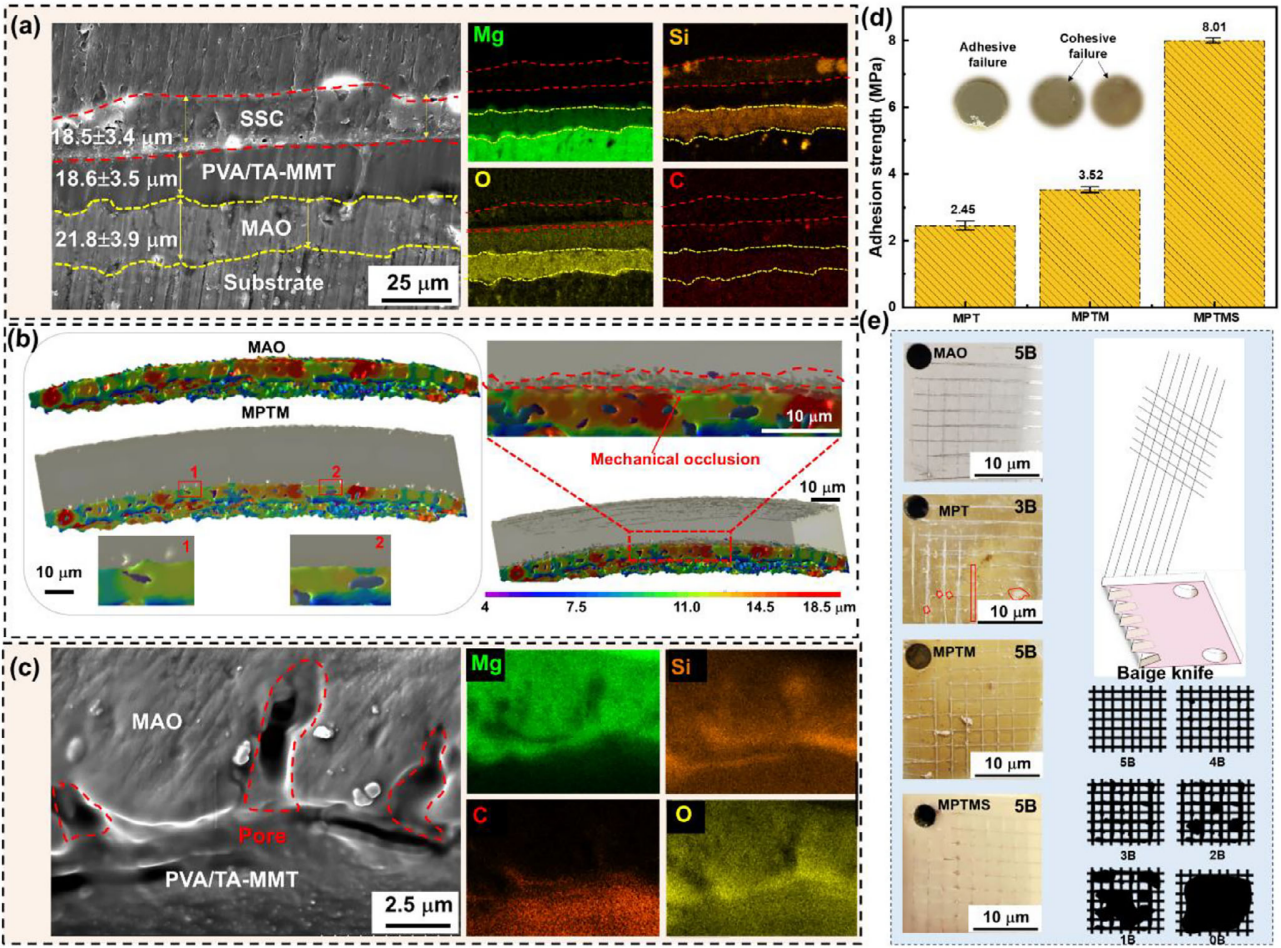
Adhesion properties of MPTMS coating. a) SEM cross‐sectional morphology and corresponding elemental distribution of MPTMS coating. b) Reconstructed 3D images of MAO and MPTM coatings. c) Magnified SEM image showing the cross‐sectional pores and corresponding elemental maps. d) Pull‐off adhesion strength and camera images showing different sample post‐tests. e) Adhesive ability of different samples.

Adhesion is a critical prerequisite for coatings, directly influencing their mechanical stability and performance in practice. Poor coating adhesion leads to delamination and the formation of defects on surface. Practical application of coatings, particularly in engineering field, involve various operating environment [30]. At high humidity, the penetration of tiny water molecules into defects of low adhesion coatings leads to accelerated failure of protective coatings. Cross‐section morphology of MPTMS reveals that there are almost no bubbles and voids at the bonded interface, and no cracks at the interface, suggesting a highly coating/substrate interface (Figure 2a). 3D interface bonding state of the composite coating was analyzed through synchrotron microtomography.

A large number of randomly distributed micropores and microcracks are visible inside and on the surface of MAO coating (Figure 2b). These micropores and microcracks on the surface can act as mechanical pinning sites and thus enhance the adhesion ability. After the hydrophilic self‐healing polymer is introduced into MAO coating, its high flowing properties in the initial state allow the polymer to completely fill the pores of MAO layer. Thermal stimulation is then applied to provide the necessary energy for the chemical reactions within the polymer, promoting cross‐linking between polymer chains. This process results in a strong bond between MAO coating and the self‐healing polymer layer. The morphology of the micropores was examined using cross‐sectional imaging, while their composition was analyzed by energy‐dispersive spectroscopy (EDS). The results reveal that the micropores are predominantly filled with materials composed of silicon (Si), carbon (C) and oxygen (O) derived from MPTM (Figure 2c). It suggests that holes on the surface of MAO coating are fully sealed through MPTM.

Adhesion was evaluated using standardized pull‐off and cross‐cut tests [31]. In pull‐off tests, adhesion strength of coatings increased progressively with addition of coating layers. A recorded adhesive strength of MPTMS is of 8 MPa (Figure 2d). Cross‐cut test results show that for MAO, MPTM, and MPTMS, the cut edge is very smooth, and the region between the cuts is not destroyed. Meanwhile, no significant fragment distribution is observed in the cut area. According to the cross‐cut adhesion test, MPTMS coating achieved a Class 5B rating, suggesting high resistance to scratching (Figure 2e).

Bioinspired high‐adhesion strategies play a critical role in improving the performance of multilayer composite coatings. Such as, a finger‐like microstructure inspired by the Phloeodes diabolicus elytra has been replicated using a simple magnetic forming process, producing an interlocking microarray with reinforced heterogeneous assembly. The resulting polyurethane‐polyimide coating achieves an adhesion strength exceeding 10.8 MPa and remains largely unaffected by corrosion exposure [32]. Similar approaches that mimic natural attachment mechanisms have led to the development of various biomimetic coatings that rely on combined non‐covalent and covalent interactions [33, 34]. In this study, we adopt a biomimetic “skin‐like” multilayer architecture to enhance coating adhesion through both mechanical pinning and interfacial chemical interactions.

The high adhesion arises from two primary mechanisms. 1) MAO layer contains numerous micropores and microcracks that provide effective mechanical interlocking sites (Figure S17). During its initial high‐fluidity stage, PVA‐TA self‐healing layer fully infiltrates these pores and forms a robust mechanical anchoring network upon solidification. 2) The unsaturated metal atoms on the surface of MMT interact strongly with hydroxyl groups in both PVA‐TA and SSC, further strengthening interfacial bonding. The high fluidity of SSC enables complete wetting of the intermediate layer, promotes chain interpenetration, and improves interfacial compatibility. Those combined mechanisms account for the excellent adhesion performance of the three‐layer MPTMS coating, which exhibits a tensile adhesion strength of ∼8 MPa and achieves 5B rating in cross‐cut adhesion tests.

### 2.3 Wetting Performance

**FIGURE 3:**
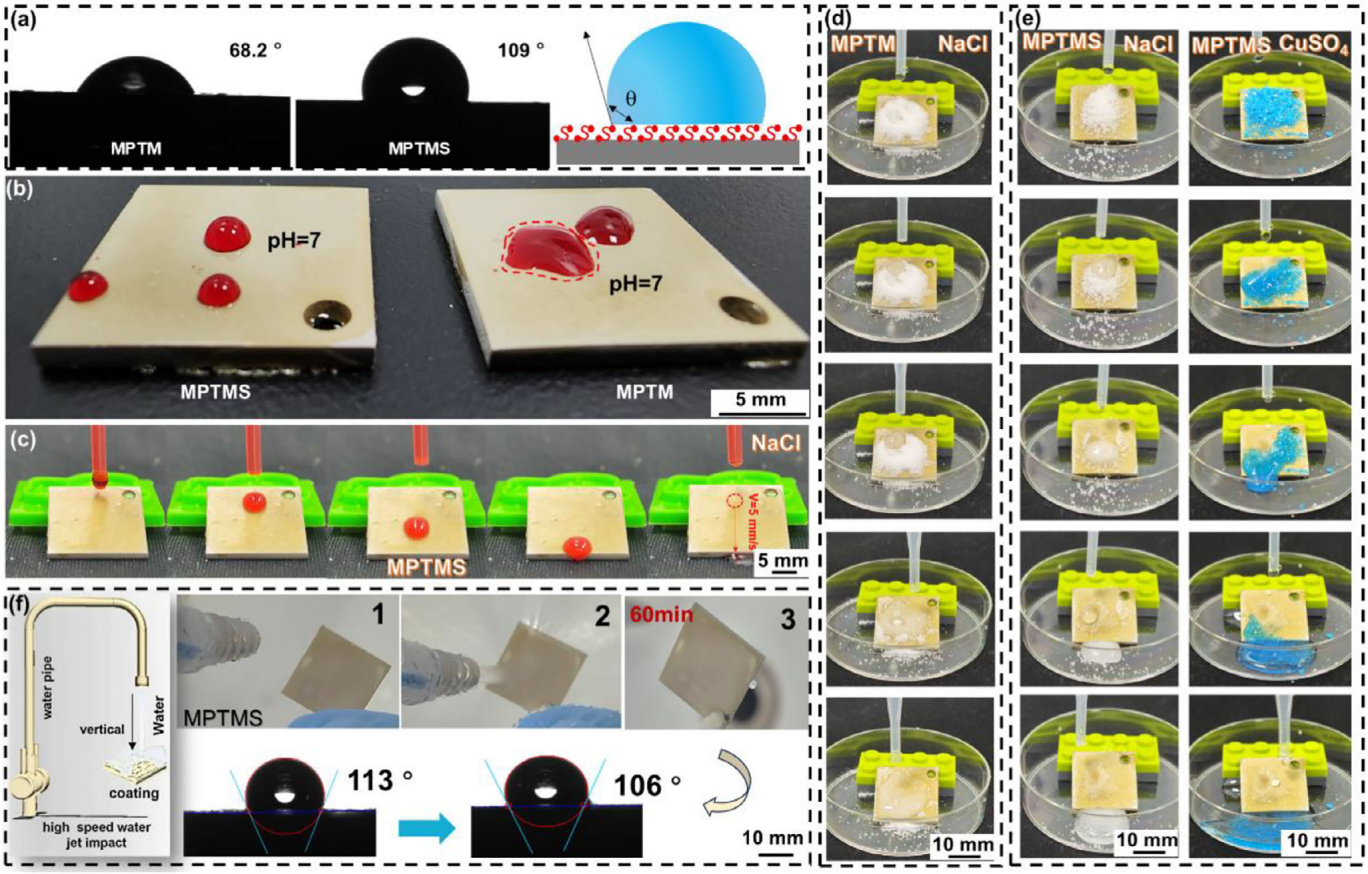
Hydrophobic, self‐cleaning, and high‐speed water impact resistance properties. a) Schematic representation of water contact angle measurement process for MPTM and MPTMS coatings. b) Images showing a neutral 3.5% NaCl droplet on sample surface. c) Sequential images depicting the sliding of a water droplet on MPTMS coating at a tilt angle of 9°. Series of images illustrating the removal of simulated contaminants from d) MPTM and e) MPTMS coatings. f) Schematic diagram of high‐speed water flow impact tests and wetting behavior of MPTMS surface under prolonged exposure to a high‐speed water flow.

Figure 3 presents hydrophobic properties of MPTM and MPTMS coatings. The static contact angle of MPTM is measured as 68.2°, indicating its hydrophilic nature. This is further evidenced by the irregular shape of liquid droplets on its surface. In contrast, MPTMS coating exhibits significant water repellency, with a contact angle of 109°. The liquid droplets on this surface formed a near half‐sphere shape, similar to those observed on SLIPS, attributed to the low surface energy of PDMS (Figure 3a,b) [35]. The slippery properties were further tested using red ink. At a 10° tilt angle, the ink droplet slid off MPTMS surface at a velocity of 5 mm/s, leaving no residue (Figure 3c). Given that protective coatings are often used in outdoor environments, resistance to contamination is crucial. When pollutants adhere to the coating surface, they can modify the local corrosive environment, leading to accelerated localized corrosion. MPTMS coating features self‐cleaning properties that significantly reduce pollutant adhesion and improve durability. A series of self‐cleaning tests were conducted on the composite coating to evaluate its effectiveness under polluted conditions.

Simulated pollutants, such as NaCl and copper sulfate powders, were uniformly distributed on the surface of the composite coating. These pollutants are easily taken away and cleaned by the sliding droplets, demonstrating the coating's self‐cleaning capability (Figure 3d,e). One of the key challenges with conventional water‐repellent coatings is balancing resistance to mechanical damage with anti‐wetting properties. This is primarily due to the loss of lubricants and damage to the micro/nanoscale roughness. High‐speed water flow impact tests were performed to assess the mechanical stability of the sample. Remarkably, the sample retained its initial hydrophobicity (106°) even with a continuous impact of 10 L/min water flow and a velocity of 0.085 m/s for 60 min (Figure 3f). This impact resistance is attributed to the strong crosslinking capabilities of epoxy resin and the silane coupling agent, along with the low surface energy and excellent hydrophobic properties of polydimethylsiloxane (PDMS).

### 2.4 Corrosion Resistance

Interlayer integration based on interface design plays a pivotal role in enhancing long‐term corrosion resistance. For example, scholars have achieved synergistic improvements in both mechanical strength and corrosion performance by constructing alternating soft polymer and hard metal oxide layers in nacre‐inspired materials [36]. Similarly, the gradient incorporation of nanoparticles within polymer matrices has been shown to enhance adhesion and corrosion resistance [20, 21]. Furthermore, leveraging the high surface affinity of poly(acrylic acid) (PAA) for iron oxides and its strong metal‐ion chelation capability can improve nanoparticle dispersion, enabling the fabrication of highly corrosion‐resistant PAA hybrid films [31]. Building on these insights, our study develops a hierarchical coating system composed of a self‐healing inner layer, an anticorrosive intermediate layer, and a hydrophobic outer layer. The integration of these layers is achieved through mechanical interlocking, controlled interfacial bonding, and crosslinking between functional groups. This structural design ensures chemical compatibility among layers and yields synergistic corrosion protection under harsh service conditions.

**FIGURE 4:**
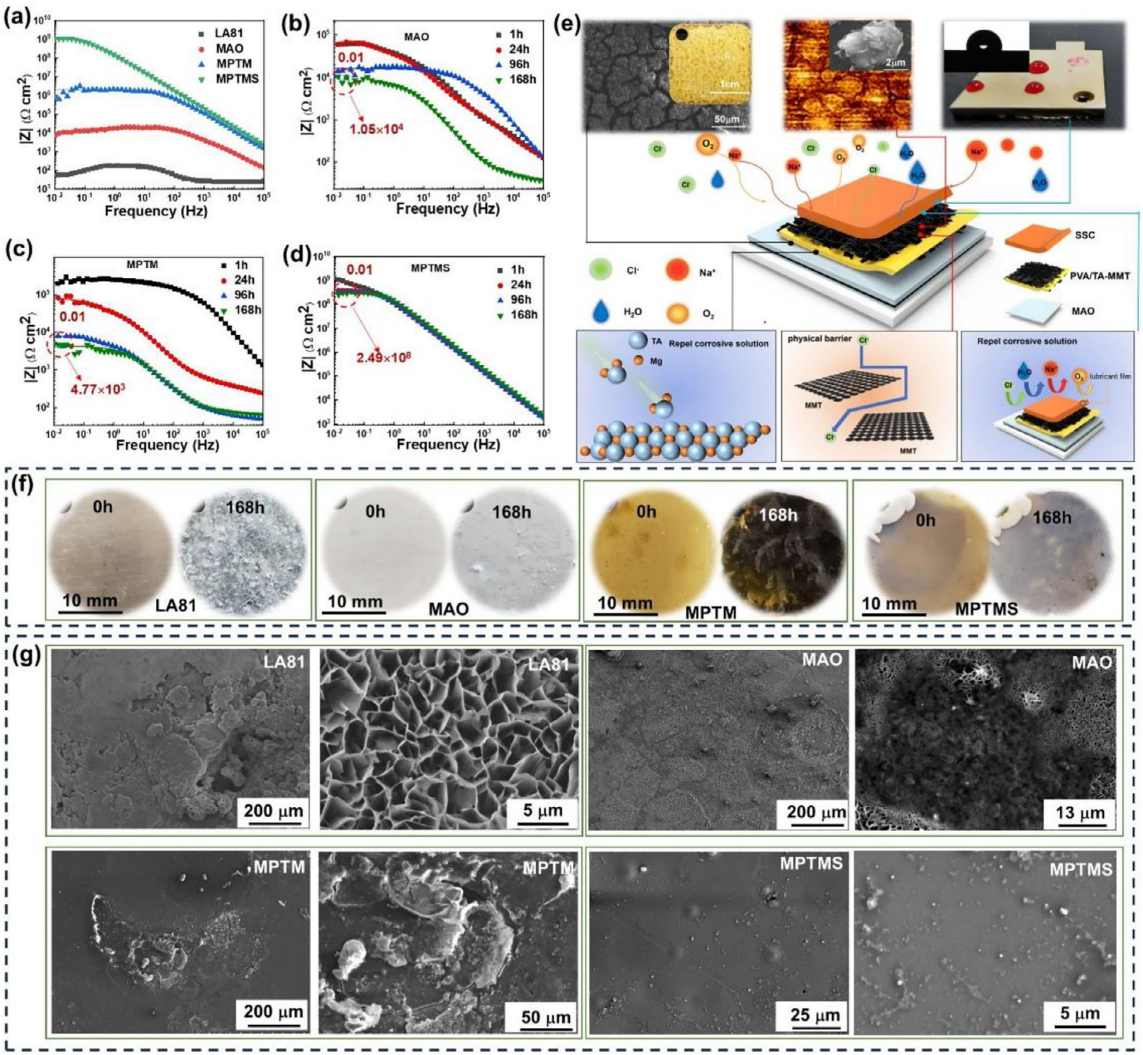
Comparison of corrosion‐resistant performance for bare Mg alloy LA81, MAO, MPTM, and MPTMS. a) Bode plots for various samples in 3.5 wt.% NaCl solution, showing their corrosion‐resistant capability. Long‐term electrochemical corrosion behavior monitored by electrochemical impedance spectroscopy: b) MAO, c) MPTM, and d) MPTMS. e) Schematic representation of the triple anti‐corrosion mechanism of MPTMS. f) Photographs, and g) SEM images showing corrosion degree of different samples after neutral salt spray tests for 168 h.

SSC layer in MPTMS not only maintains stable anti‐wetting properties but also minimizes contact with corrosive media, thereby improving corrosion resistance and durability [20, 21]. Additionally, the integration of MPTMS with flake MMT, known for its high barrier properties, further prolongs the diffusion pathways for corrosive agents [37]. Consequently, this composite coating exhibits outstanding anti‐corrosion performance, even in highly corrosive environments. Corrosion resistance of the sample was evaluated using a combination of neutral salt spray (NSS) testing and electrochemical measurements. MPTMS yields the biggest value of |Z|_0.01_(1.0 × 10^9^) and capacitive arc compared with other samples, showing strong corrosion resistance (Figure 4a; Figure S18).

EIS data of samples were analyzed using DRT in 3.5 wt.% NaCl solution [38]. The equivalent circuit model, as illustrated in Figure S19, demonstrates that incorporating SSC layer increases the peak impedance of the coating by four orders of magnitude compared to the coating without SSC. The composite coating displays three distinct peaks, each corresponding to a constant phase element influenced by parallel capacitance and resistance, which suggests the presence of three different electrochemical processes. At low frequencies, the time constant is dominated by the double‐layer capacitance at the coating‐substrate interface and the charge transfer resistance associated with corrosion of Mg–Li alloys. The well‐separated peak observed at mid‐frequency indicates that MAO and PTM undergo a single electrochemical process, reflecting a unified coating system.

Stability of the coating samples was further evaluated in neutral 3.5% NaCl aqueous solution to assess their performance in a saline environment. As shown in Figure 4b–d and Figures S20 and S21, MPTM initially offered mild protection to MAO coating. However, over time, the performance of this coating perishes. After 168 h, its protective capability is inferior to that of the single‐layer MAO coating. In contrast, MPTMS maintained an impedance modulus of |Z|_0.01_ (2.5 × 10^8^) after 168 h of immersion, which is substantially higher than the |Z|_0.01_ values of the MAO coating (1.1 × 10^4^) and the self‐healing hydrophilic polymer (4.8 × 10^3^). It demonstrates that SSC coating effectively protects the self‐healing hydrophilic polymer from moisture damage, preserving its stability and corrosion resistance even under harsh water conditions.

Figure S21 illustrates EIS behavior of various coatings and reveals the following trends. MAO coating exhibits the lowest impedance (1.8 × 10^4^ Ω·cm^2^), indicating limited corrosion protection. MAO‐TA coating shows higher impedance (3.9 × 10^4^ Ω·cm^2^), confirming the effective chelation between TA and the MAO layer. MAO‐PVA coating demonstrates a moderate improvement (3.9 × 10^4^ Ω·cm^2^), primarily due to the sealing effect of PVA. In contrast, LA81‐PVA/TA coating shows the lowest corrosion resistance (7.5 × 10^3^ Ω·cm^2^), likely resulting from poor surface compactness. Macroscopic observations revealed that during curing, the reactive Mg–Li substrate interacted with the solvent, generating gas bubbles that produced local defects and reduced coating uniformity. PVA‐TA‐modified MAO coating achieves the highest impedance (≈1 × 10^5^ Ω·cm^2^), attributed to synergistic hydrogen‐bonding and chelation effects that enhance barrier performance. Collectively, these results demonstrate that PVA and TA contribute distinct yet complementary functions in improving corrosion resistance, and that their integration within the hybrid coating provides a substantially greater protective effect than either component alone. The corrosion protection mechanism results from the combined effects of three key factors: the strong barrier properties of MAO, and MMT, the hydrophobic characteristics of SSC layer, and the chelation between tannic acid molecules and Mg ions produced during the corrosion process. These elements work together to enhance the overall protective performance (Figure 4e).

The major challenge in applying self‐healing hydrophilic polymers in engineering is their sensitivity to environmental moisture. Excessive moisture can lead to swelling and diffusion of these polymers, ultimately degrading their performance. To assess coating durability under harsh conditions, salt spray testing is employed. This test simulates a severe, high‐humidity corrosive environment. Coatings that successfully endure 7 days salt spray tests are typically deemed suitable for practical engineering application. MPTM and MPTMS specimens were exposed to a harsh simulated salt spray environment. After 24 h, MPTM is compromised by the corrosive water vapor, leading to a noticeable color change and the appearance of visible corrosion pits. After 168 h, the hydrophilic polymer showed evident signs of delamination. In contrast, MPTMS, which features an additional hydrophobic coating, exhibited no significant corrosion even after exposure for 168 h (Figure 4f,g).

The coatings maintained a high level of hydrophobicity, with the contact angle decreasing only slightly from 109° to 101.6° after exposure 168 h in 3.5% NaCl solution (Figure S22). Such a minor reduction demonstrates the excellent durability of the MPTMS coating. Furthermore, optical microscopy images confirmed that the surface morphology of the hydrophobic layer remained largely unchanged, indicating that the coating retained its physical texture after prolonged immersion. Collectively, these results confirm that the hydrophobicity of the coating remains stable over time, supporting its sustained contribution to corrosion protection.

### 2.5 Self‐Healing Performance

**FIGURE 5:**
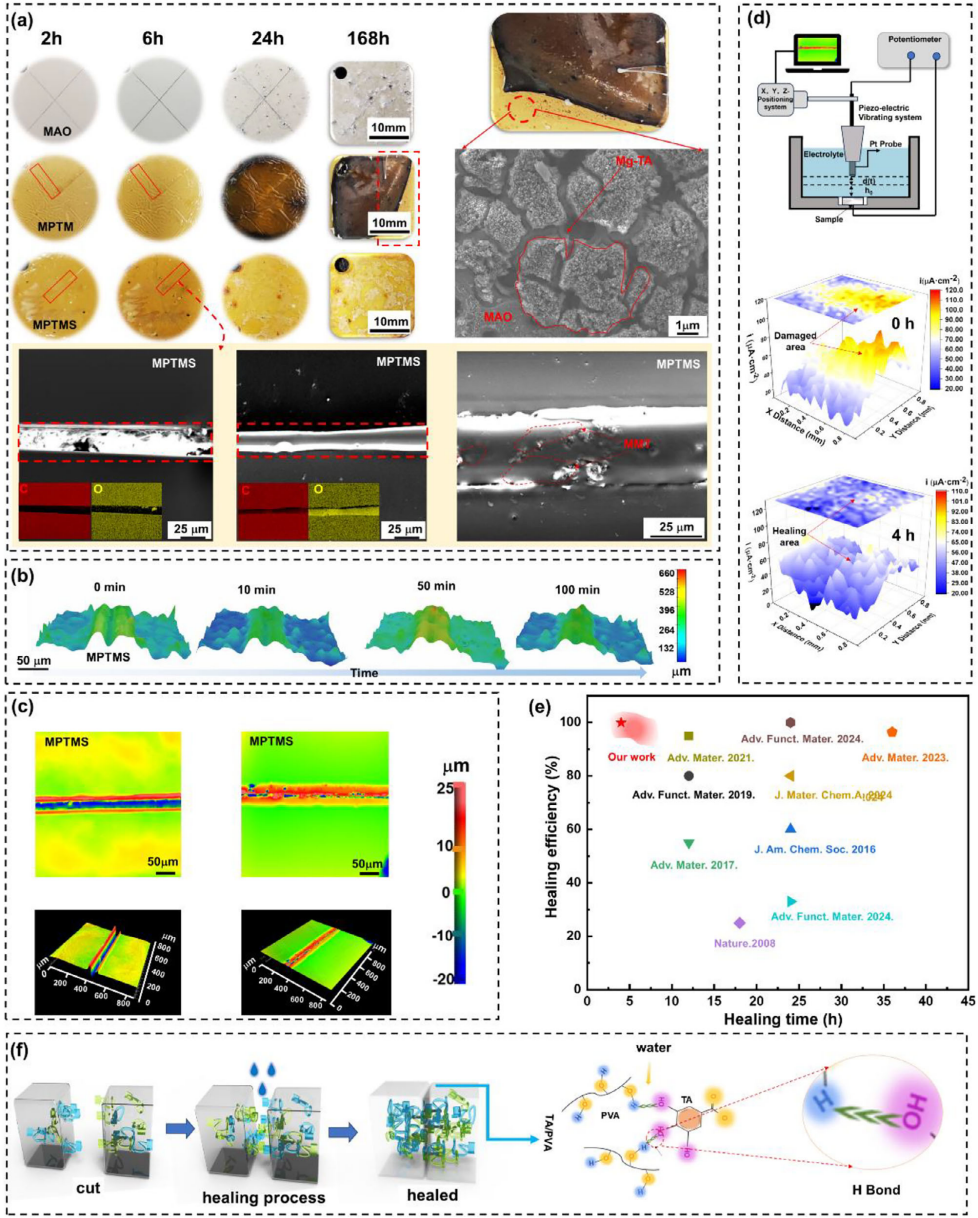
Self‐healing properties of MPTMS coatings. a) Self‐healing process of MPTMS coating observed using optical microscopy and SEM following extended exposure to neutral salt spray testing. Evolution of surface damage on MPTMS, monitored using b) laser confocal and c) 3D microscopy, after applying 3.5% NaCl solution to scratched areas. d) SVET technique capturing potential maps of damaged MPTMS coating in 3.5 wt.% NaCl solution. e) Schematic illustration showing the self‐healing mechanism driven by hydrophilic polymer diffusion and hydrogen bonding in an aqueous environment. f) Comparison of healing time and efficiency of MPTMS coating with reported self‐healing materials.

Hydrophilic self‐healing polymers are rich in hydroxyl groups that form strong intermolecular hydrogen bonds. When the material is damaged, these hydrogen bonds can reform, allowing the polymer to effectively repair physical damage. The coating surfaces are deliberately scratched with a sharp blade and then exposed to a high‐humidity salt environment (Figure 5a). After 6 h, MPTM demonstrated effective recovery, with surface scratches nearly disappearing. In contrast, MPTMS, owing to its hydrophobic layer, retained fewer water molecules at the damaged sites. Although the scratches on this coating are almost invisible to naked eyes after 6 h, its repair efficiency is lower compared to that of MPTM. Microscopic analysis showed that scratches on the surface of MPTMS were approximately 40 µm wide. After undergoing the self‐healing process, these scratches are completely covered by a new layer of coating, demonstrating exceptional repair capabilities. This finding highlights the coating's effective self‐healing performance at the microscopic level. In contrast, as moisture increased, the MPTM began to swell, causing a color change from yellow to black and leading to extensive delamination and signs of aging (Figure S23). After 168 h, MPTMS retained its original yellow color and exhibited no significant signs of aging, indicating effective resistance to moisture‐induced corrosion. These results suggest that MPTMS offers a substantial improvement in extending the service life of MPTM.

After detachment of the coating layer, a pale‐yellow film remained on the surface. Microscopic analysis revealed that this film exhibited a cracked soil morphology, consistent with classic conversion coatings. Water diffusion weakened the hydrogen bonds between the hydroxyl groups of PVA and the phenolic hydroxyl groups of TA [15]. Additionally, TA's hydroxyl and carboxyl groups readily interacted with the aqueous solution, increasing the concentration of TA molecules near the hydrophilic self‐healing polymer. These TA molecules chelated with magnesium ions released during corrosion, ultimately forming an eco‐friendly conversion coating for magnesium through the use of TA solution.

As shown in Figures S24 and S25, to further evaluate the role of SSC coating (MPTMS), we compared EIS responses of scratched samples with and without SSC protection (MPTM). For samples containing the SSC layer, the impedance modulus gradually increased after damage, from approximately 3 × 10^3^ to 10^4^ Ω·cm^2^, and remained stable at this level even after 168 h of immersion. In contrast, samples without SSC exhibited an initial rapid impedance recovery, reaching nearly 10^4^ Ω·cm^2^ within 60 min. However, their impedance subsequently declined with extended immersion, dropping to 812 Ω·cm^2^ after 24 h. This deterioration suggests that prolonged exposure to aqueous environments disrupts the hydrophilic polymer's hydrogen‐bond network, thereby reducing self‐healing efficiency. The presence of SSC layer effectively mitigates this degradation by minimizing direct contact between the electrolyte and the underlying hydrophilic polymer, thereby preserving the integrity of the hydrogen‐bonding network. Consequently, MPTMS coating exhibits superior post‐damage corrosion resistance compared with MPTM coating.

MPTMS exhibited exceptional self‐healing capabilities, attributed to dynamic hydrogen bonding and water‐induced diffusion. As shown in Figure 5b,c, surface scratches of 50 µm are nearly completely healed within 100 min in a humid environment. From the microprobe technique perspective, SKP and SVET was used for understanding the in situ self‐healing process. SVET 3D mapping of the current density measured above MPTMS containing the scratch region are displayed in Figure 5d. SVET reflects ionic fluxes from corrosion activity, and yellow regions are detected as local corrosion. Mechanical damage to the surface exposed the Mg–Li alloy substrate directly to 3.5% NaCl solution. Initially, severe corrosion is observed in the scratched areas of the coating. However, after 4 h of immersion, the localized corrosion diminished, and the self‐corrosion current density became uniformly distributed across the surface. The color contrast in the mapping indicated no visible corrosion area. This observation suggests that corrosion did not occur, revealing that the coating effectively achieved self‐healing. SKP was utilized to analyze the surface volta‐potential distribution. The peak values in the volta potential distribution map highlight active regions within the scratch gaps. Initially, the potential difference along the scratches is pronounced, indicating a greater susceptibility to localized corrosion. Over time, this potential difference became more uniformly distributed across the scratches, and the peaks eventually disappeared (Figure S26).

The self‐healing mechanism of this system resembles the synergistic healing effect observed in dual‐layer structures within aqueous environments, as described by Qi's competitive diffusion model [15]. This process also involves the moisture‐triggered reconstruction of hydrogen bonds between hydroxyl groups in hydrophilic polymers, such as TA and PVA. This mechanism is illustrated in Figure 5e. In this study, MPTMS exhibits rapid damage repair within a very short period. When the cut surfaces of PTMS are immersed in a water environment for 4 h, the healing efficiency remained at 100%, which is considerably higher than those previously reported self‐healing coatings (Figure 5e,f) [14, 39, 40, 41, 42, 43, 44]. Our system achieves nearly 100% self‐healing efficiency in a water environment for 4 h (Figure 5), which we compare with previously reported water‐sensitive dynamic bond systems, such as hydrogels healed by hydrogen bonds [45] and borate bonds [46]. Most of these reported systems exhibit significant degradation of healing efficiency in aqueous environments. In contrast, our three‐layer structure maintains rapid self‐healing capability even under salt spray and water immersion, demonstrating distinct advantages in durability and environmental stability.

**FIGURE 6:**
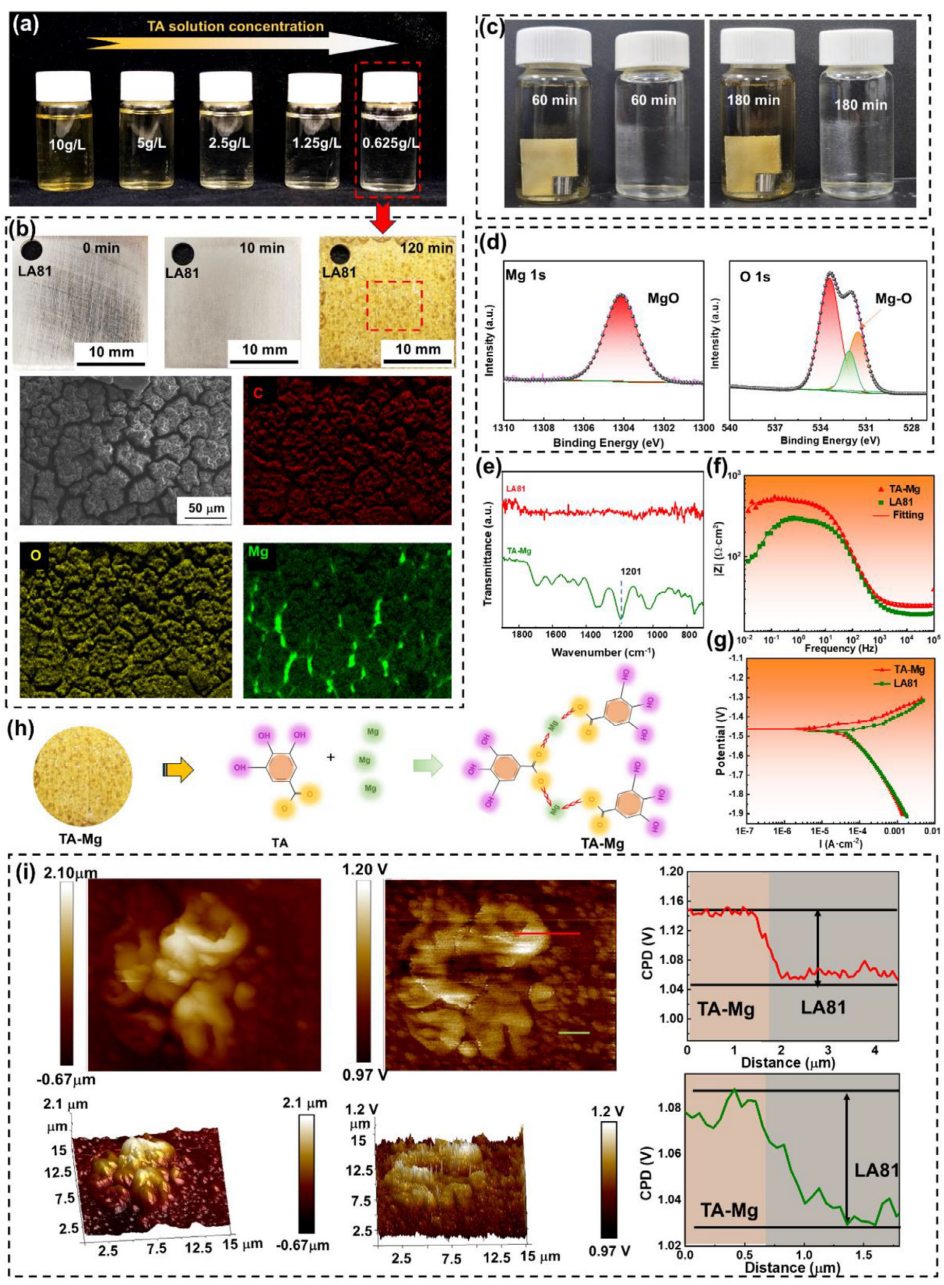
Impact of tannic acid on corrosion performance of Mg–Li Alloy. a) Digital images of TA aqueous solutions at varying concentrations. b) Digital and SEM images of Mg–Li alloy after immersion in 0.625 g/L TA solution for different durations. c) Digital images showing MPTM immersed in ultrapure water for various time periods. d) XPS spectra of tannic acid Mg conversion film. e) Raman spectra of Mg–Li alloy substrate before and after immersion in tannic acid solution. f) Electrochemical impedance spectroscopy and g) potentiodynamic polarization curves for Mg–Li alloy and tannic acid Mg coatings. h) Schematic representation of the formation mechanism of the tannic acid Mg conversion film on Mg–Li alloy surface. i) KPFM images of tannic acid Mg coating.

To elucidate effects of TA concentration on chemical conversion coating and corrosion resistance of Mg–Li alloy, aqueous TA solutions with varying concentrations were prepared (Figure 6a). At a concentration of 0.625 g/L with a 120 min treatment, a stable TA conversion coating is obtained, as shown in Figure 6b. Notably, the solution color gradually turned from pale yellow to yellowish brown as the concentration of TA increased. Increasing the TA concentration significantly reduce the coating preparation time from 120 to 20 min (Figure S27). A control experiment was conducted to observe the release of TA from the hydrophilic self‐healing polymer in an aqueous solution (Figure 6c). After 180 min of immersion, the solution transitioned from colorless and transparent to light yellow, closely matching the color of 10 g/L tannic acid solution. Under these conditions, Mg–Li alloys readily form magnesium–tannate conversion coatings. Component analysis of the conversion coating, also illustrated in the Figure 6d,e,h, demonstrates that TA, a plant‐derived polyphenol, is rich in hydroxyl (─OH) and aldehyde (─CHO) groups [47]. Mg ions coordinate with these active groups through ionic or coordinate bonds. The positively charged Mg ions attract the negatively charged TA molecules, leading to the formation of a stable complex.

Furthermore, electrochemical data and Kelvin probe force microscope (KPFM) were conducted for evaluating corrosion‐resistant capability of TA conversion coating [48]. TA conversion coating yields the biggest E_corr_ (−1.46 V) and the smallest *i*_corr_ (2.5 × 10^−5^A⋅cm^−2^) compared with Mg‐Li alloy LA81 (9.9 × 10^−5^ A⋅cm^−2^), reveal a strong corrosion resistance. In EIS plot(Figure 6f,g), the impedance value significantly increases from 84.2 Ω⋅cm^2^ for LA81 to 3.7 × 10^2^ Ω⋅cm^2^ for TA conversion coating, showing that the conversion coating can significantly protect Mg–Li substrate from corrosion. It can be seen from Figure 6i that the Volta potential of Mg–Li matrix is negative, and the Volta potential on the surface of coating gradually shifts to the positive direction. The high work function of the conversion coating necessitates greater energy to extract electrons from the surface, reducing its susceptibility to corrosion compared to Mg–Li alloy LA81 substrate. Furthermore, TA conversion coating demonstrates a significant electrochemical potential difference relative to Mg–Li alloy. This potential difference provides effective cathodic protection, thereby enhancing corrosion resistance of Mg–Li alloy LA81.

Due to the reversible hydrogen bonding between PVA and TA, water‐induced polymer diffusion, and the recurrent formation of Mg‐TA conversion layers, the coating can undergo multiple cycles of damage‐repair, analogous to the repeatable healing behavior of natural skin.

## 3 Conclusions

This study proposes a multifunctional, biomimetic anti‐corrosion self‐healing coating that combines commercially available reagents with a simple MAO and scalable doctor blade coating process. The collaborative gradient design of this coating integrates multiple functions, including wettability, self‐repair, and strength, addressing the conflicting demands between hydrophilic self‐healing polymers and corrosion protection in water environments. The innovative approach of this biomimetic skin opens new pathways for the broad applications of hydrophilic self‐healing polymers in a range of engineering services.

## 4 Experimental

### 4.1 Materials and Reagents

Mg–Li alloy LA81 plates (dimension 20 × 20 × 1.5 mm^3^) were used in this study. The average composition of LA81 alloy is Al 1.04 wt.%, Li 8.40 wt.%, and Mg in balance. Sodium hydroxide (NaOH, AR), sodium chloride (NaCl, AR), ethyl acetate (C_4_H_8_O_2_, AR), sodium silicate (Na_2_SiO_3_, AR), and potassium fluoride (KF, AR) were purchased from Aladdin (Shanghai, China). Polydimethylsiloxane with a viscosity of approx. 50 mPa·s at 25°C was acquired from Sihai Chemical Technology Co., Ltd. (China). Polyvinyl alcohol (PVA, M.W. = 47 000 g mol^−1^) and TA (M.W. = 1701 g mol^−1^) were purchased from the commercial agency (Macklin biochemical Co., Ltd., Shanghai, China). Montmorillonite (MMT, 99%), epoxy resin (E44, epoxy equivalent content: 210−230g⋅mol^−1^), and silane coupling agent (KH550, AR) were purchased from Alibaba Group Holding Limited (China). Commercially available hydrophilic silica nanoparticles (average size of 20 nm) were obtained from Shanghai Zhong Te Nano Technology Co., Ltd. (China). All the aforementioned chemical agents were used without further treatments.

### 4.2 Preparation of MPTMS Composite Coatings

The MPTMS was prepared using a three‐step process. This involved a base layer with a micro‐nano porous structure, an intermediate layer composed of a self‐healing polymer, and a top layer of a solid‐like slippery coating with hydrophobic properties, as schematically illustrated in Figure 1. First, Mg–Li alloy LA81 pieces were progressively mechanically ground with SiC sandpapers from grit 100 to 2000, then ultrasonically cleaned in ethanol at ambient temperature to remove contaminations and rapidly dried with a blow dryer. Pretreated Mg–Li alloy LA81 substrate was anodized using a commercially available MAO equipment. The coating was deposited using an AC power supply at 300 V and 500 Hz for 3 min. Electrolyte solution contained 8 g/L of NaOH, 10 g/L of Na_2_SiO_3_, and 5 g/L of KF. MAO coated samples (designated as MAO hereafter) were ultrasonically cleaned in deionized water, and dried in an air flow.

In terms of preparation of MPTM, in a three‐neck round‐bottom flask, 5 g of TA and 4 g of PVA were combined with a mixture of 35 mL deionized water and 30 mL of ethanol. This mixture was stirred mechanically at 250 r/min. After heating at 100°C for 1 h, a PVA/TA solution was obtained. Then, MAO coating was completely immersed in the PVA‐TA solution for 1 min, followed by curing in a vacuum oven at 70°C for 20 min. Next, a 10 mg/mL^−1^ MMT suspension was obtained by dispersing 1 g of MMT powders into 100 mL DI water under vigorous stirring. Then, 30 mL of PT solutions were added into a mixture of 40 mL of MTM suspension (10 mg/mL^−1^) and 20 mL of ethanol to form PTM solution. Subsequently, 5 mL PTM solution was carefully pipetted onto PT coating surface and evenly spread using a scraper blade set to a height of 200 µm. The liquid film was dried at 70°C for 20 min to obtain self‐healing intermediate layer.

In terms of preparation of MPTMS, hydrophilic silica nanoparticles (0.3 g), silane coupling agent (5.0 g), and polydimethylsiloxane (10.0 g) were dissolved in 30 mL ethyl acetate solution under mechanical stirring speed of 600 r/min for 24 h to obtain Solution α. Epoxy resin (5.0 g) was then dissolved in 30 mL of ethyl acetate for 12 h to form Solution β. Afterward, Solutions α and β were mixed and stirred for 6 h to obtain SSC solution. Then, MPTM coating was completely immersed in SSC solution for 1 min, followed by curing at 25°C to form the hydrophobic barrier top layer.

### 4.3 Surface Characterization

Surface and cross‐sectional morphology, and elemental compositions of the samples were characterized by field‐emission scanning electron microscopy (FE‐SEM, SU5000, HITACHI, Japan) equipped with energy‐dispersive X‐ray spectrometry (EDS Oxford Ultim Max). Surface roughness and 3D morphology were determined through atomic force microscopy (AFM, Bruker JPK Nanowizard 4XP, German), and 3D surface optical profilometry (NewView9000, ZYGO Corporation, America), respectively. Surface wettability–related water contact angles (CA) and sliding angles (SA) were all measured by the OCA50 system (DataPhysics, Germany). Surface chemical composition was unveiled through Fourier transform infrared spectrophotometry (FT‐IR, Nicolet IS50 FTIR Spectrometer, Thermo Nicolet, America) and Raman spectroscopy (DXR Microscope Raman Spectrometer, ThermoFisher Scientific, America) with a laser wavelength of 532 nm. Phase composition was identified by X‐ray diffractometry instrument (XRD, D8 Advance, Bruker, German) at a grazing angle of 4° with Cu Kα radiation within a range of 5−90°. X‐ray photoelectron spectroscopy (XPS, ESCALAB250Xi, ThermoFisher Scientific, America) was employed to explore chemical state of O1s, C1s, Al, and Si 2p in MPTM, O1s, C1s, and Si 2p in MPTMS samples.

### 4.4 Mechanical Tests

Adhesion strength of different coatings was measured by using an adhesion tester (TC‐A Coating adhesion tester, China) according to the ASTM D4541standards. An aluminum aalloy was adhered to the surface of the coatings using epoxy adhesive (Kafuter K‐9761). Data were gathered by detaching the alloy at 0.2 MPa s^−1^. An average value was calculated from five different test points on each coating. Meanwhile, the adhesion strength of the different MPTMS coating was determined using the internationally standardized cross‐cut tests (ASTM D3359‐09). To determine the interface state between MAO and MPTM coatings, 3D image of MAO coating and interface of MAO coating and MPTM were obtained by synchrotron radiation X‐ray tomography performed in BL13HB beamline of Shanghai Synchrotron Radiation Facility (SSRF). MPTMS sample for 3D characterization was a cylinder with a diameter of ca. 1 mm. Two thousand projections were collected for each tomographic scan over 180°. Synchrotron X‐ray energy and exposure time were 25 keV and 800 ms, respectively, with a spatial resolution of 0.65 µm/pixel (Figure S28).

### 4.5 Corrosion Performance Characterization

An electrochemical workstation (CS2350M, CORRTEST, China) was employed to detect the corrosion resistance of samples. A typical three‐electrode cell system was performed as follows: the sample (with an exposed surface area of 1 cm^2^) as the working electrode, a Pt plate as counter electrode, and a saturated calomel electrode (SCE) as reference electrode. Firstly, open circuit potential (OCP) was recorded to allow the system to be stabilized in 3.5% NaCl solution and then electrochemical impedance spectroscopy (EIS) was carried out in a frequency range of 10^5^ to 10^−2^ Hz with an amplitude of 10 mV around OCP. Zsimpwin software was used to implement the fitting analysis on EIS results. Potentiodynamic polarization curves were measured with a scanning rate of 1 mV/s to ensure accuracy of the data, and the corrosion potential (E_corr_) and corrosion current density (*i*_corr_) can be estimated through Tafel extrapolation. Three independent samples were tested for each condition to ensure reproducibility.

### 4.6 Self‐Healing Properties

Self‐healing capability of MPTMS coating was explored through the scratch method. To initiate the test, cut‐through scratches were introduced into MPTMS sample using a knife blade. The incisions were extended to a depth of approximately 30 µm to fully penetrate the coating and expose the underlying Mg–Li alloy substrate. Subsequently, the scratched sample was immersed in a water environment (3.5 wt.% NaCl solution) to facilitate the healing of the damages. Morphology of the damaged areas was examined using optical microscope, 3D surfaces optical profilometry and SEM.

Local corrosion behavior was monitored using the scanning vibrating electrode technique (SVET) and scanning Kelvin probe (SKP), both implemented with a Princeton VERSA SCAN instrument. The samples, which had an exposed surface area of 1 cm^2^, served as the working electrode. Artificial scratches were introduced, and immersion experiments were conducted to assess the self‐healing property. Artificial scratches were introduced using a sharp blade, creating grooves approximately 20 mm long and 0.5–0.6 mm wide, with depths sufficient to expose the underlying M‐gLi alloy LA81 substrate. A Pt‐Ir electrode probe with a diameter of 10 µm was positioned on the designated working electrode surface, maintaining a distance of approximately 100 µm. Scans were carried out perpendicular to the length of the cut edge, within a square scanning area of 800 µm × 800 µm. The scanning step in both X‐axis and Y‐axis directions was set at 10 µm. During immersion in a 3.5 wt.% NaCl solution for 2 h, the samples were continuously monitored. To calculate the current density, denoted as “*i*” and measured in mA·cm^−^^2^, the formula *i* = ‐kΔ*V*/Δ*r* was employed, where “k” represents the solution conductivity (16.5 ± 0.2 mS·cm^−1^), Δ*V* stood for the measured potential difference in the amplitude of Δ*r* (30 µm in the experiment). The scratched regions for specimens immersed in 3.5 wt.% NaCl solution for 0 and 4 h were subsequently measured and analyzed.

## Funding

This work was financially supported by National Natural Science Foundation of China [52371005, 52534009], the Liaoning Revitalization Talents Program (XLYC2403182), Liaoning Province Science and Technology Plan Joint Program (2024JH2/102600032) and the Fundamental Research Funds for the Central Universities (DUTZD25220). E. G. thanks Xiaomi Foundation for support. X.C. is grateful to the financial support by the Australian Research Council (ARC) through the Discovery Scheme (DP240101430). The authors thank the BL13HB of Shanghai Synchrotron Radiation Facility (https://cstr.cn/ 31124.02.SSRF.BL13HB) for beamtime access and technical support.

## Conflicts of Interest

The authors declare no conflicts of interest.

## Supporting information




**Supporting File**: advs73543‐sup‐0001‐SuppMat.docx.

## Data Availability

The data that support the findings of this study are available in the supplementary material of this article.
