# Supplementary material for: Skin‐Inspired Design of Self‐Healing Coatings Integrating Interface‐Liquid Repellency and Corrosion Resistance
Source: Adv Sci (Weinh). 2026 Feb 10;13(18):e21067. doi: 10.1002/advs.202521067 (PMC13042887; doi:10.1002/advs.202521067)
Supplement: Supplementary file 1 — Supporting File: advs73543‐sup‐0001‐SuppMat.docx. [file ADVS-13-e21067-s001.docx]

**Skin-inspired Design of Self-healing Coatings Integrating Interface-liquid Repellency and Corrosion Resistance**

*Bingzhi Li, Bingce Liu, Enyu Guo^*^, Zhihao Zhou, Yibo Ouyang, Xiao-Bo Chen ^*^, Huijun Kang, Zongning Chen, Tongmin Wang^*^*

Bingzhi Li, Bingce Liu, Enyu Guo, Zhihao Zhou Huijun Kang, Zongning Chen, Tongmin Wang

Key Laboratory of Solidification Control and Digital Preparation Technology (Liaoning Province), School of Materials Science and Engineering, Dalian University of Technology, Dalian 116024, PR China

E-mail: [eyguo@dlut.edu.cn](mailto:eyguo@dlut.edu.cn); tmwang@dlut.edu.cn

Enyu Guo, Huijun Kang, Zongning Chen, Tongmin Wang

Ningbo Institute of Dalian University of Technology, Ningbo 315000, PR China

Xiao-Bo Chen

Department of Mechanical, Manufacturing and Mechatronics Engineering, School of Engineering, RMIT University, Melbourne 3000, Victoria, Australia

xiaobo.chen@rmit.edu.au

Yibo Ouyang

Department of Mechanical Engineering, Harbin University of Science and Technology, Rongcheng 264300, PR China


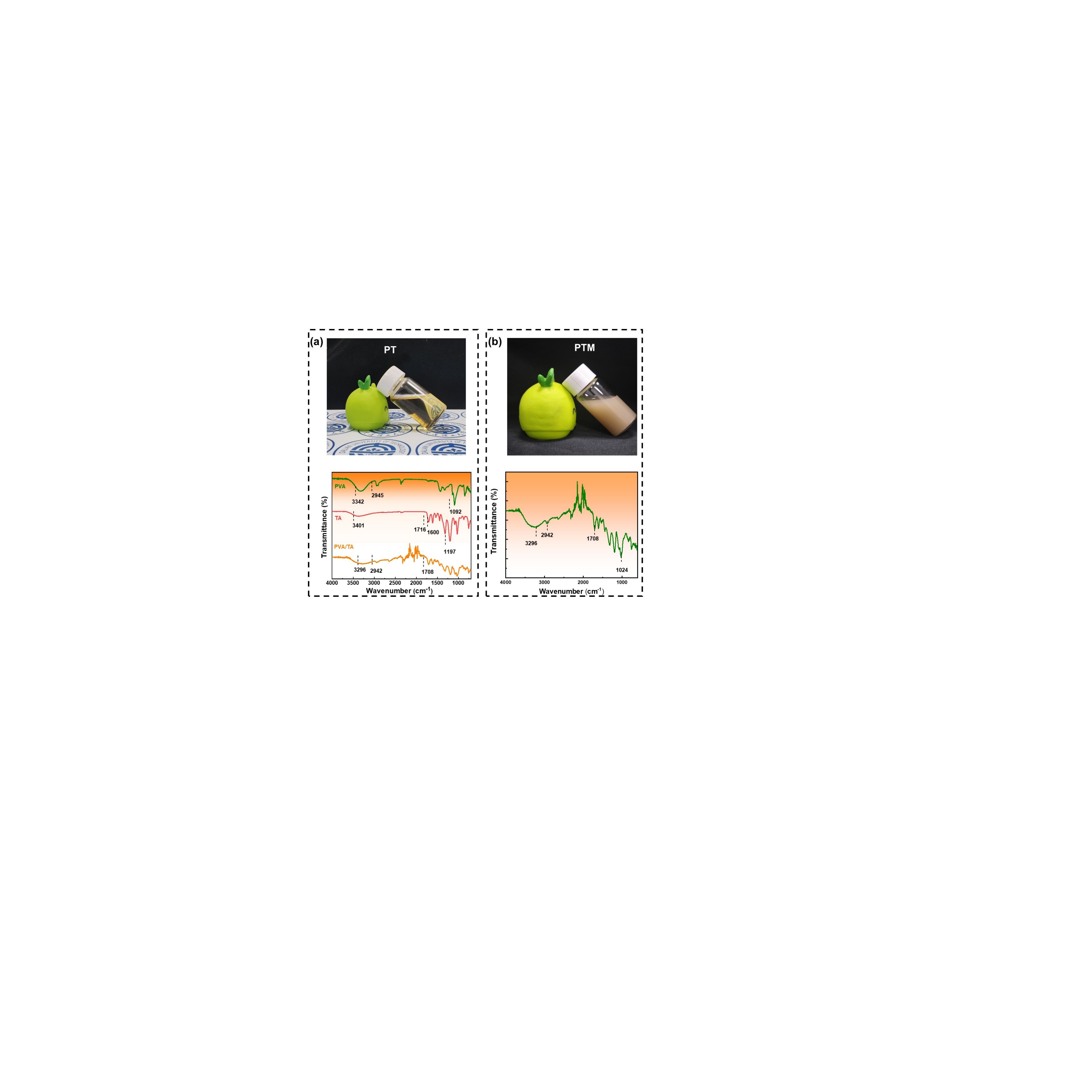


**Figure S1.** Optical photograph of initial state and FTIR spectra of a) PT hydrophilic self-healing polymer, and b) PTM hydrophilic self-healing polymer.


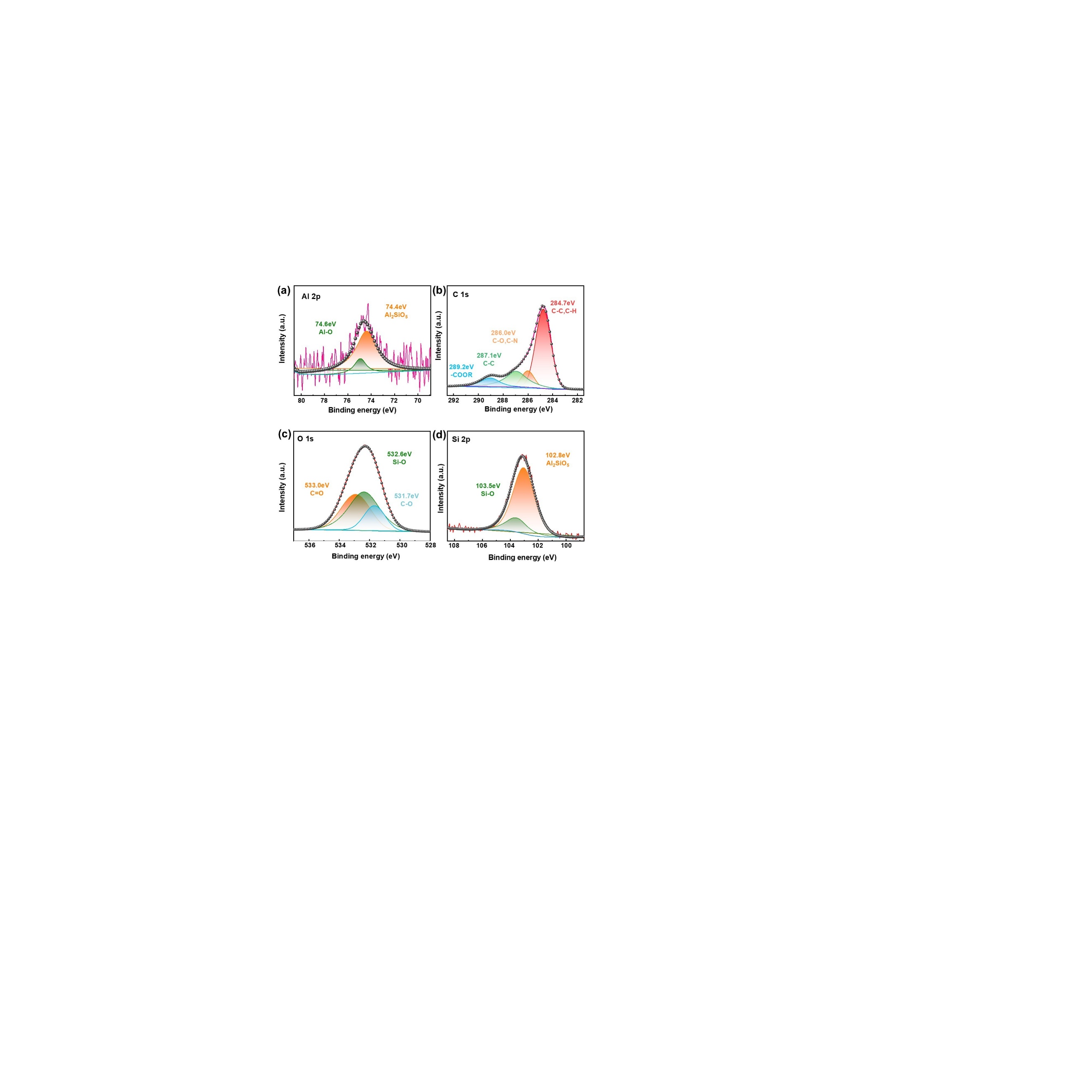


**Figure S2.** High-resolution XPS spectra for a) Al 2p, b) C 1s, c) O 1s, and d) Si 2p in MPTM coating.


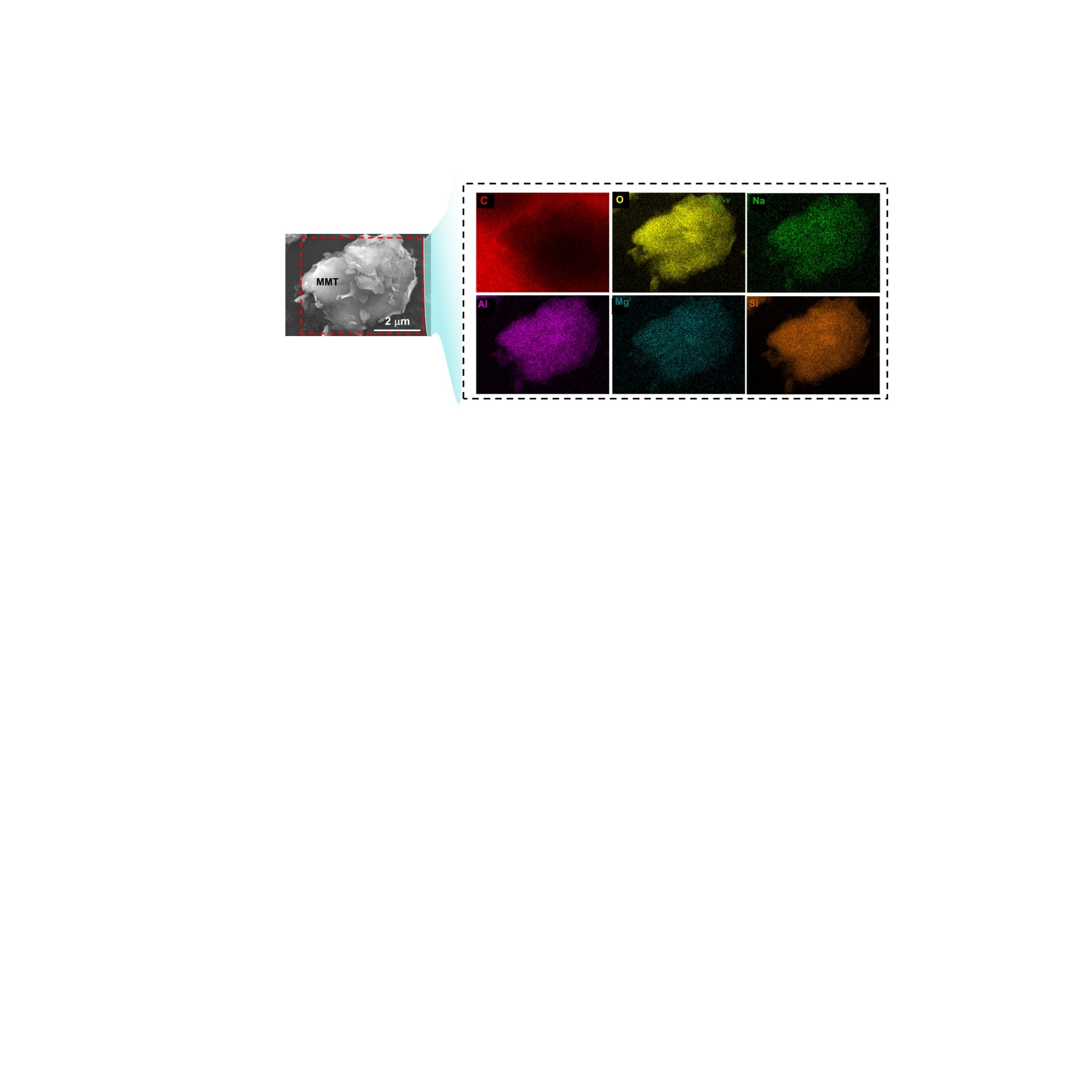


**Figure S3**. SEM micrograph and elemental mapping of MMT.


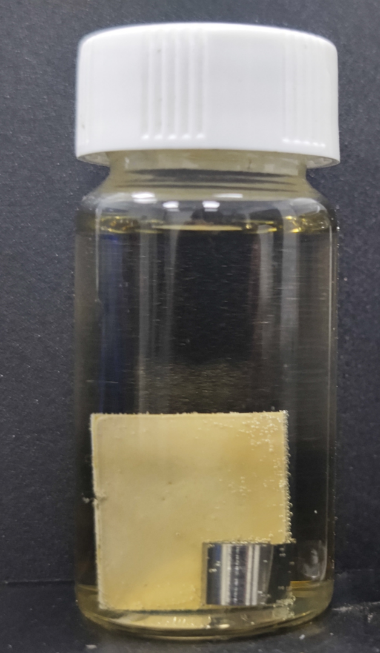


**Figure S4.** Stability of hydrophilic self-healing PTM in water solution.


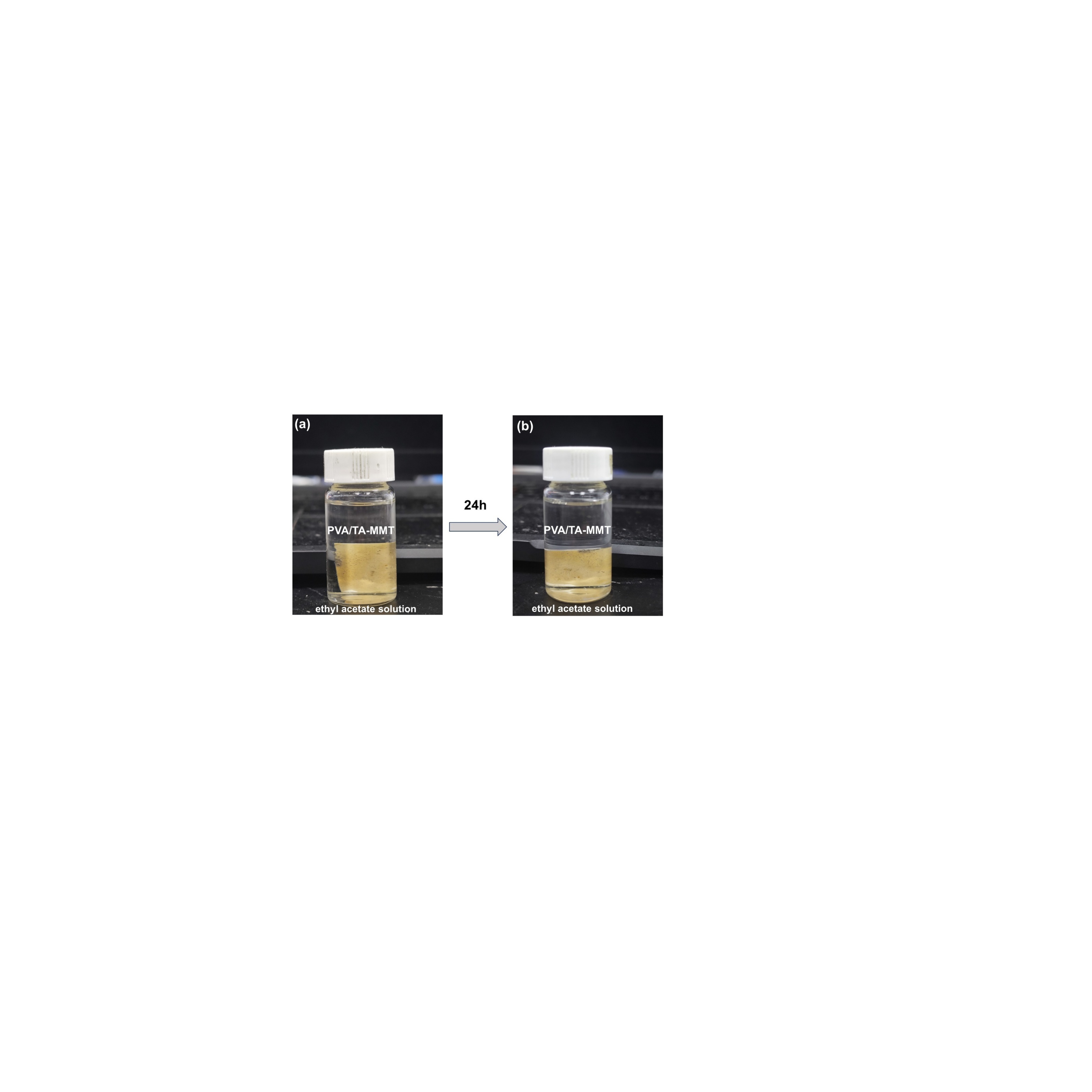


**Figure S5.** Stability of hydrophilic self-healing PTM in ethyl acetate solution. a) Initial state of hydrophilic self-healing PTM in ethyl acetate solution, b) State of hydrophilic self-healing PTM after soaking in ethyl acetate solution for 24 h.


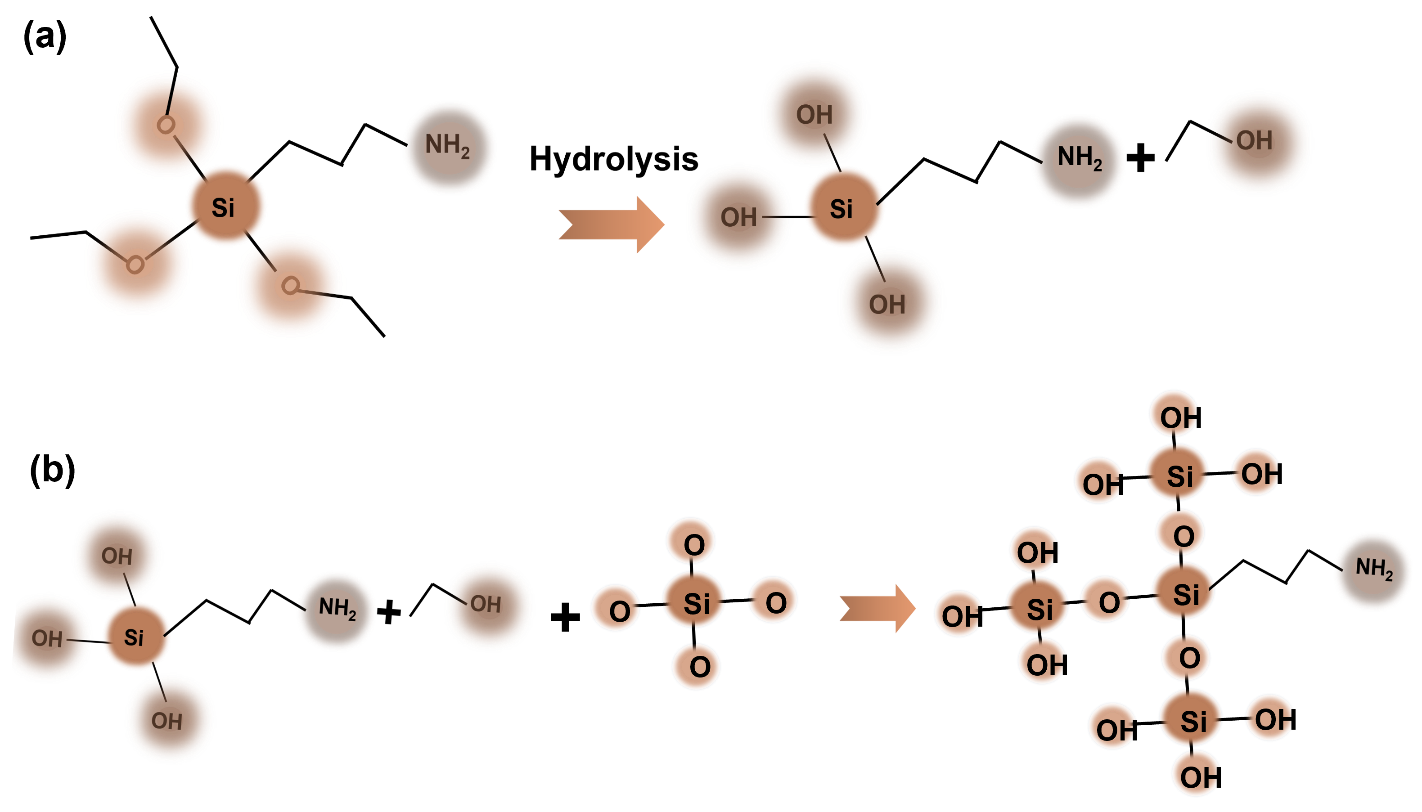


**Figure S6**. a) Hydrolysis of KH550 silane coupling agent, and b) condensation reaction between KH550 and hydrophilic nano-silica.


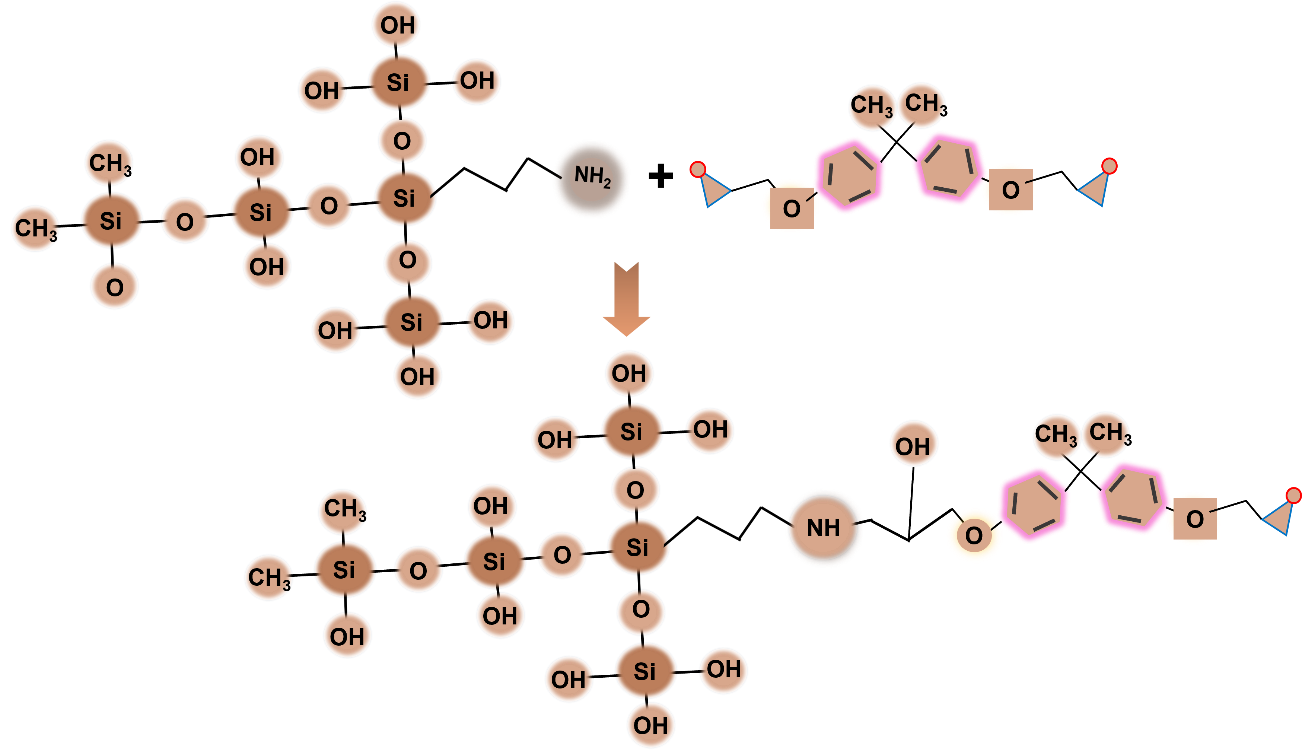


**Figure S7.** Schematic illustration of crosslinking reaction between epoxy groups and KH550-modified hydrophilic nano-silica loaded with polydimethylsiloxane.


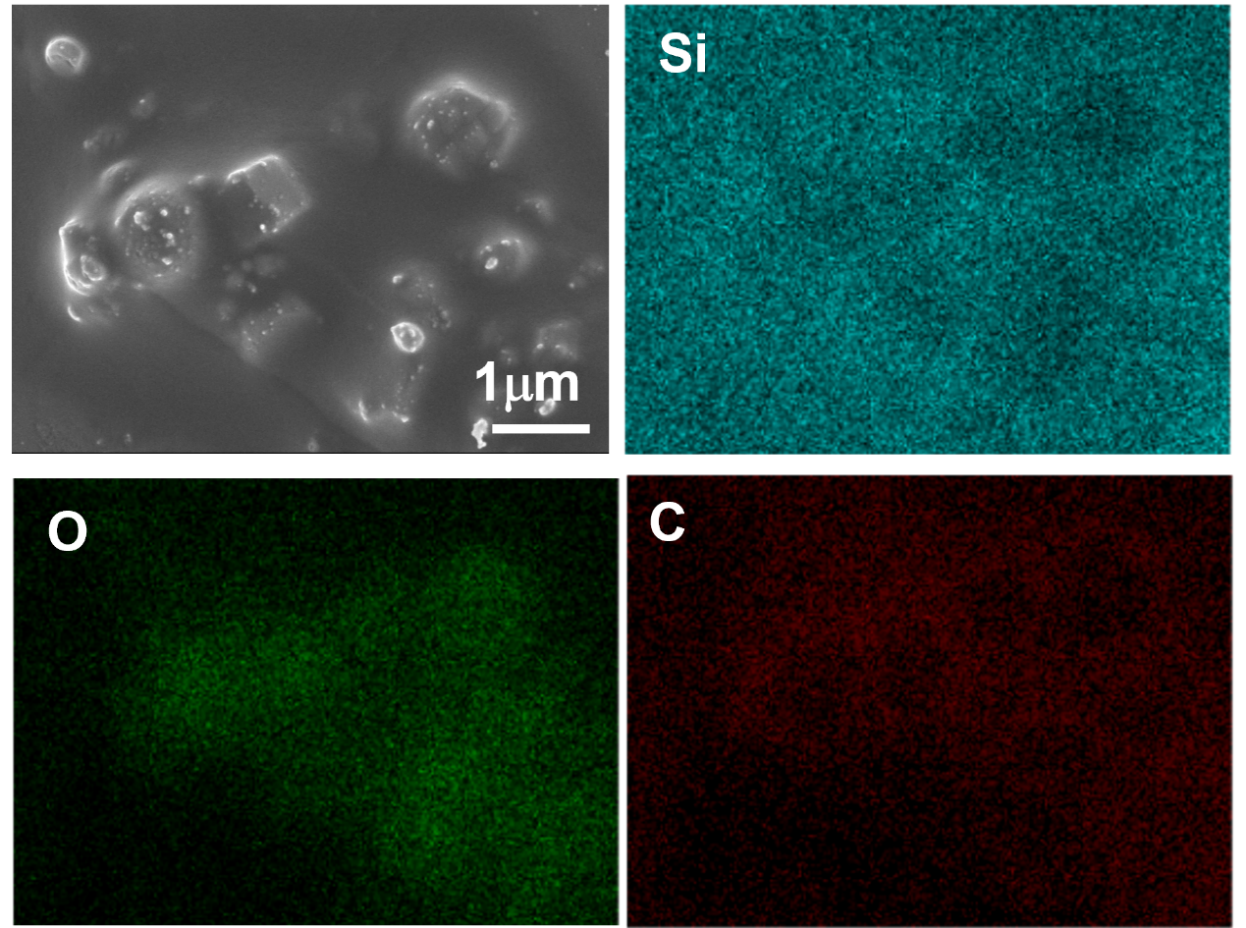


**Figure S8.** Cryo-electron microscopy (Cryo-EM) image and EDS of SSC in a fluid state.


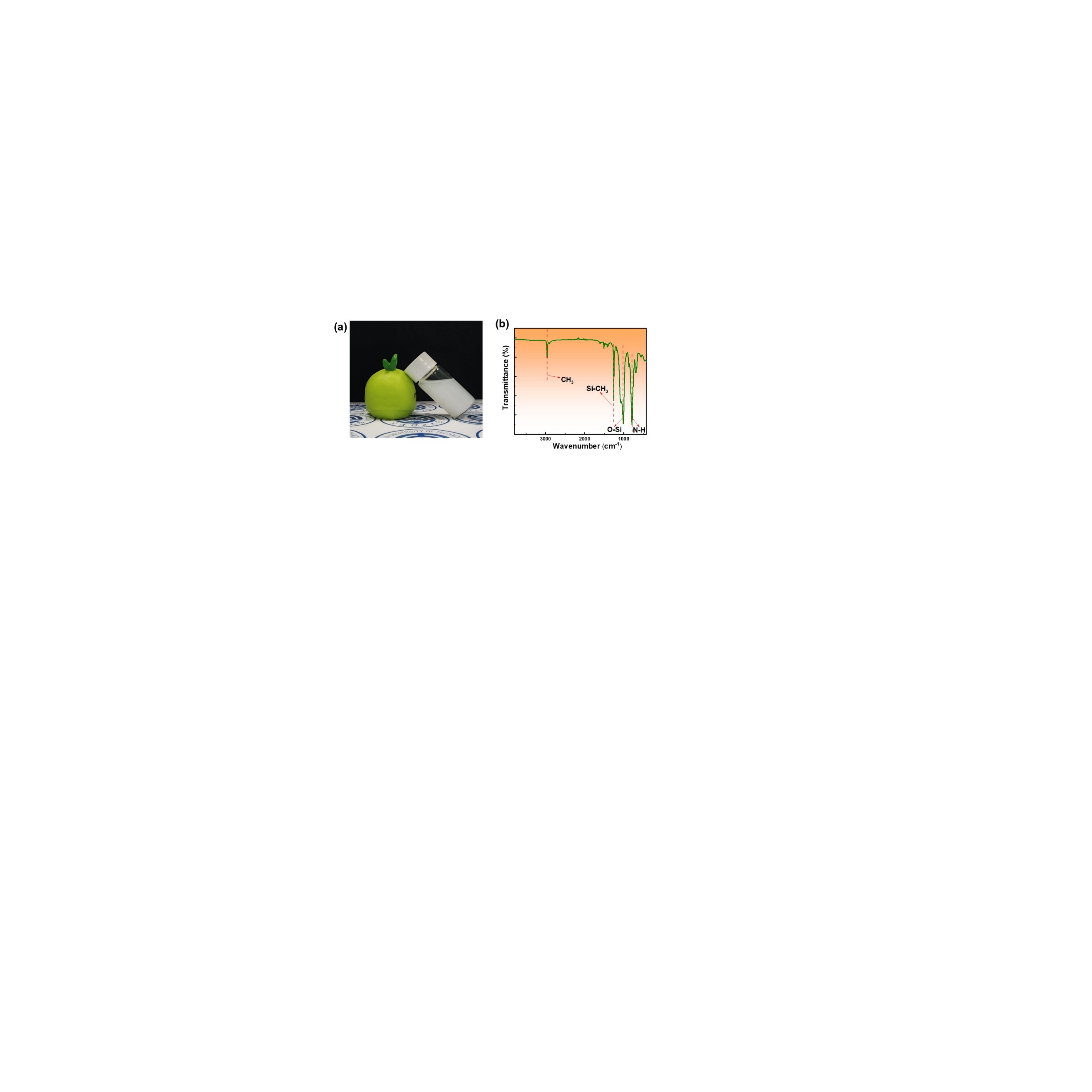


**Figure S9.** Morphology and composition of SSC coating. a) Macroscopic morphology image, and b) FTIR spectra of SSC.


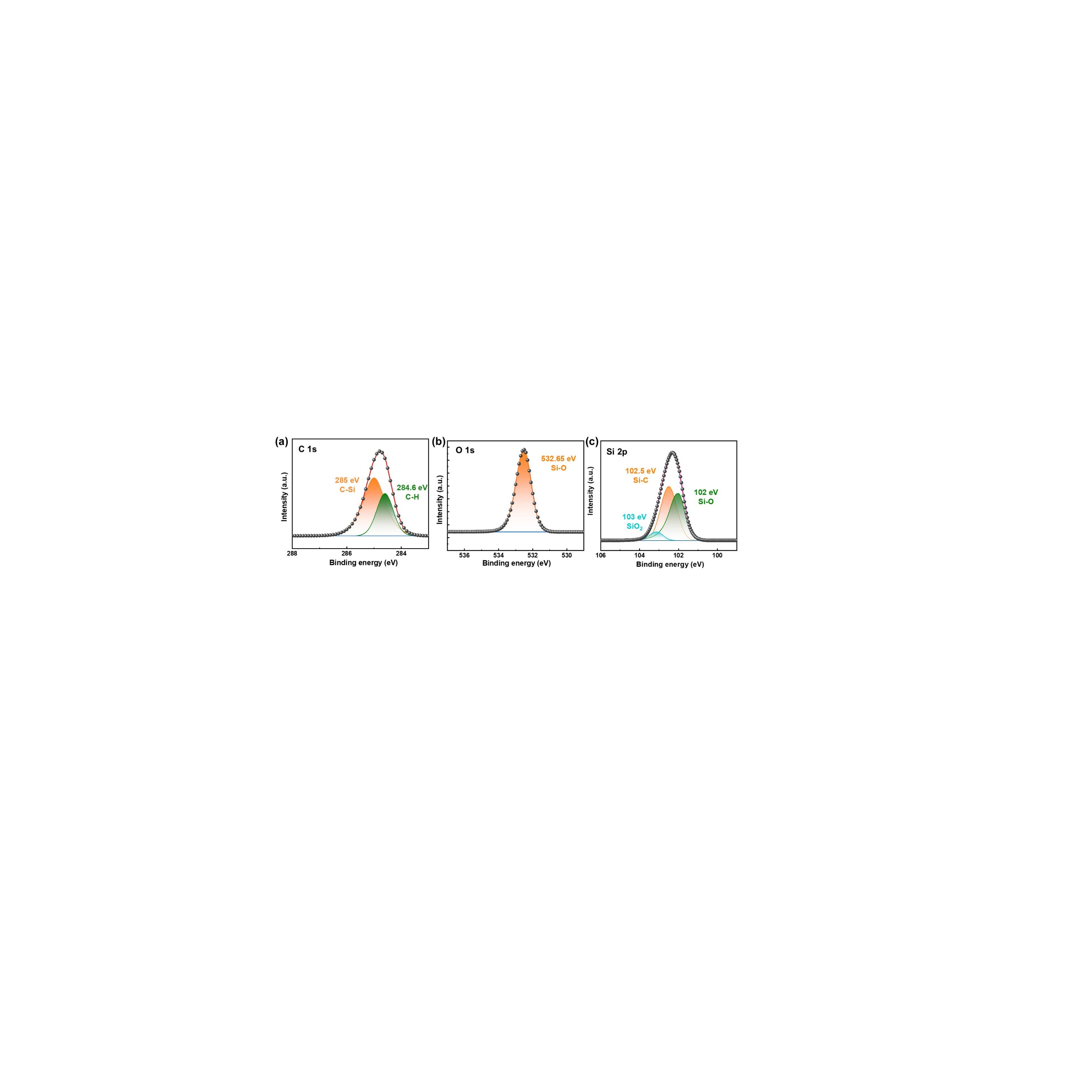


**Figure S10.** High-resolution XPS spectra of MPTMS coating for a) C 1s, b) O 1s, and c) Si 2p.


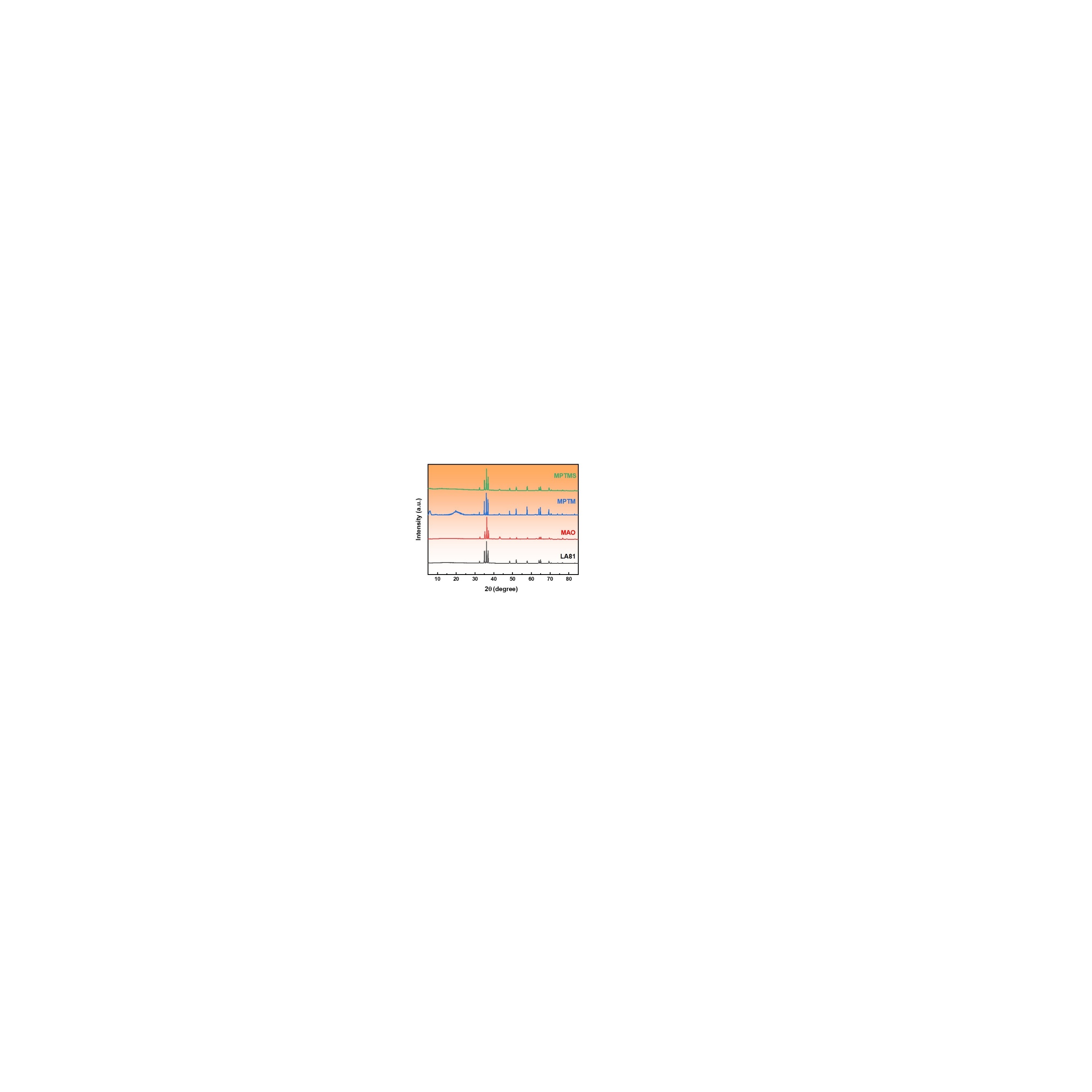


**Figure S11.** XRD patterns of LA81, MAO, MPTM, and MPTMS.


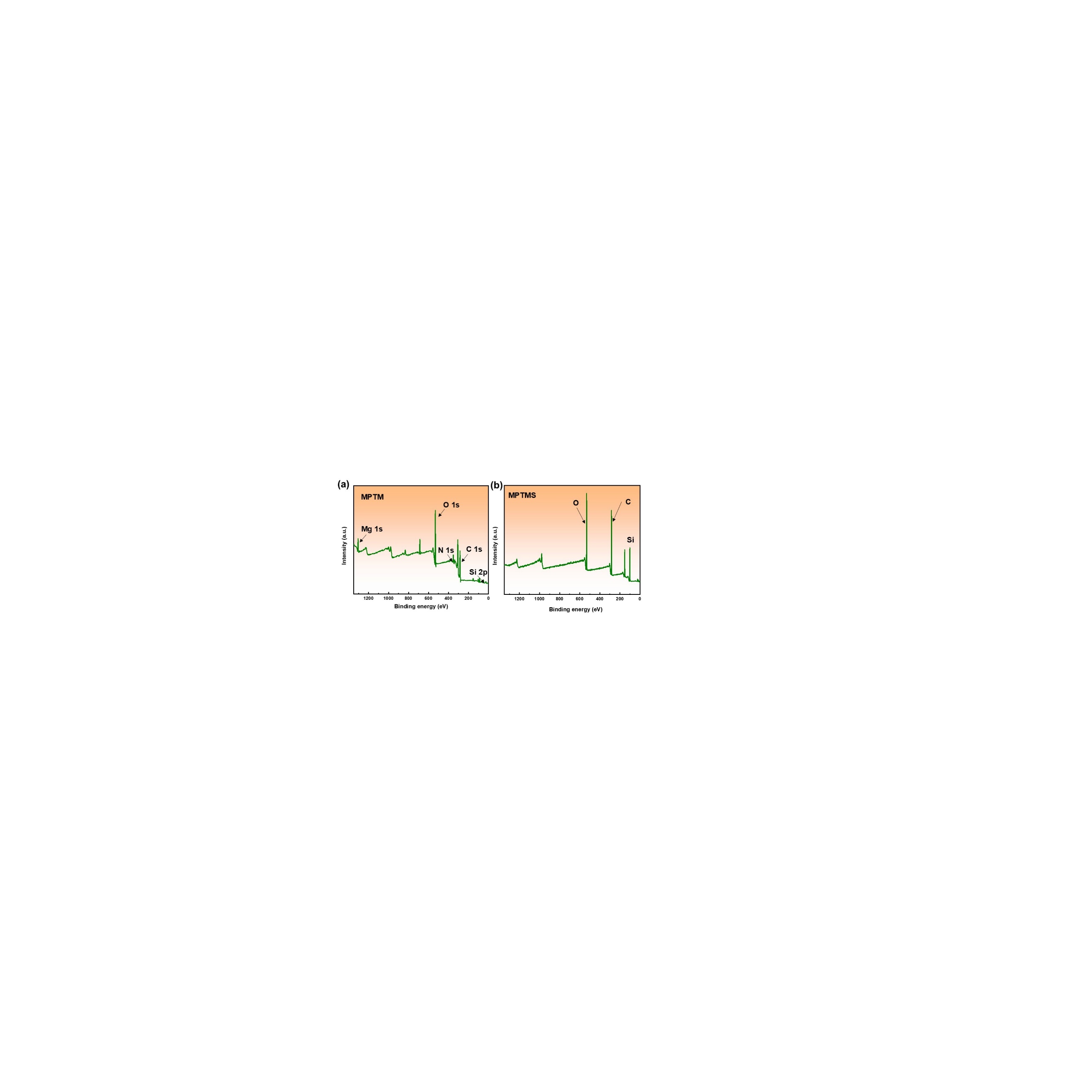


**Figure S12.** Full XPS survey spectra of a) MPTM and b) MPTMS.

**
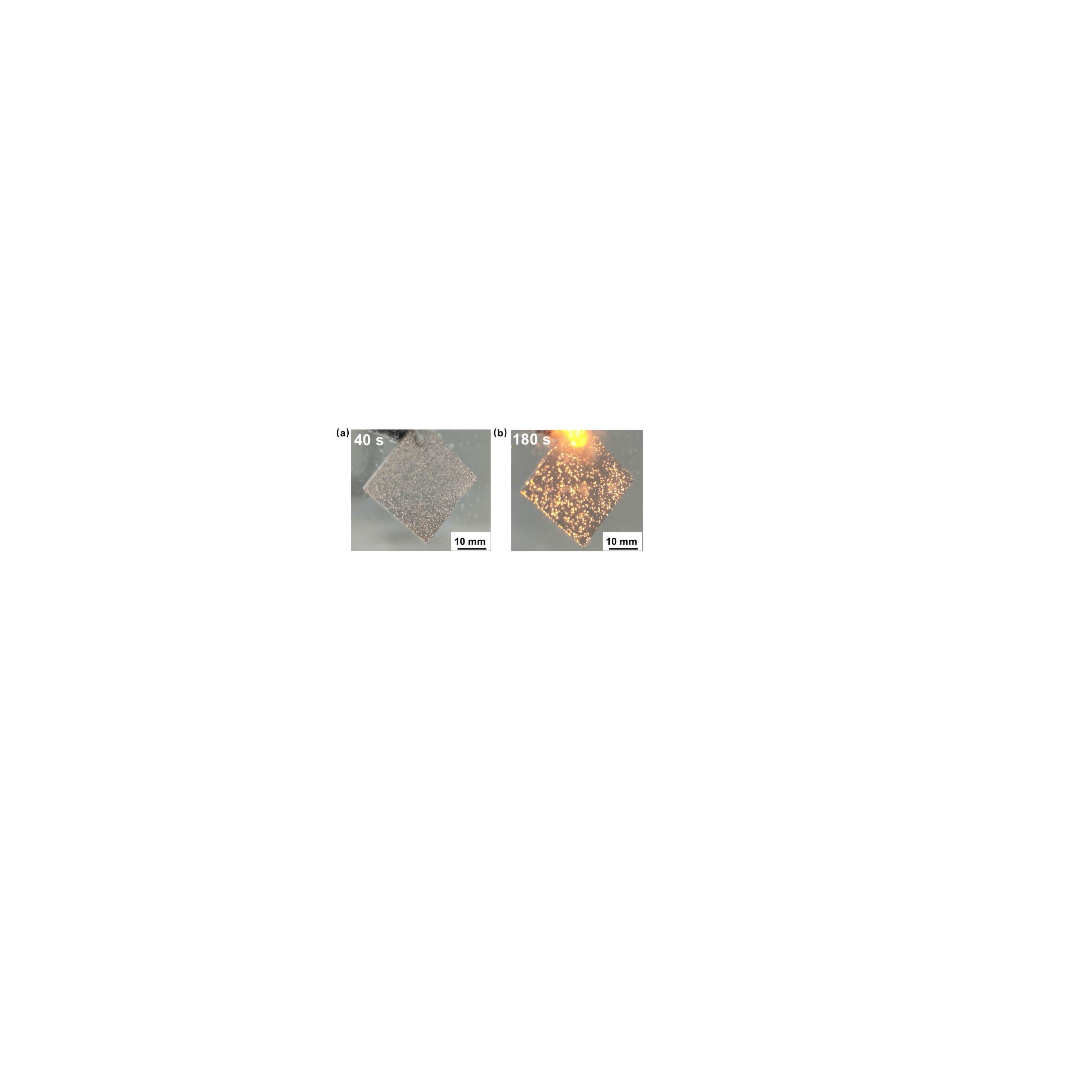
**

**Figure S13.** Spark discharge during the preparation of MAO coating with a) 40 s, and b) 180 s.


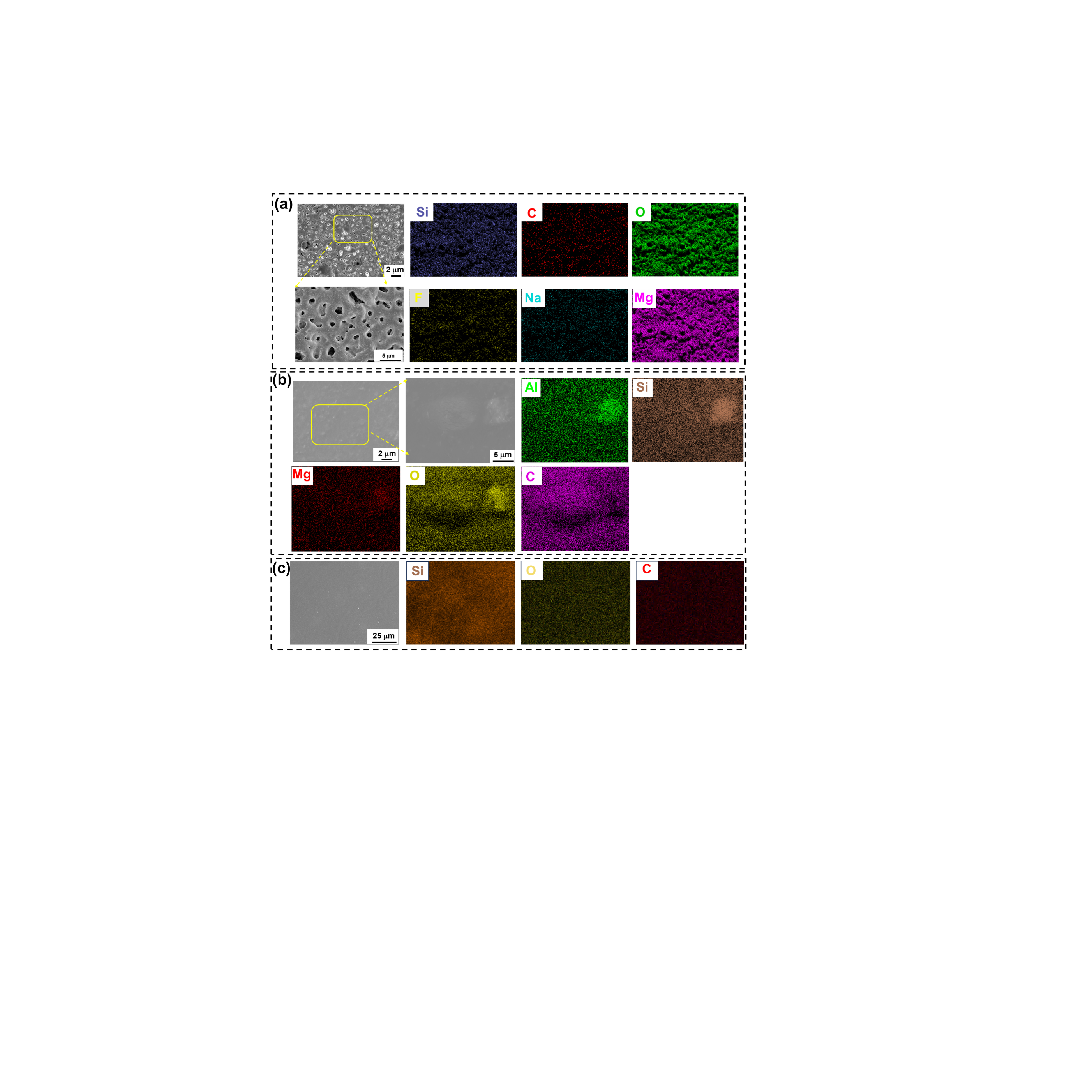


**Figure S14.** SEM images and EDS elemental distribution maps of a) MAO, b) MPTM, and c) MPTMS coatings.


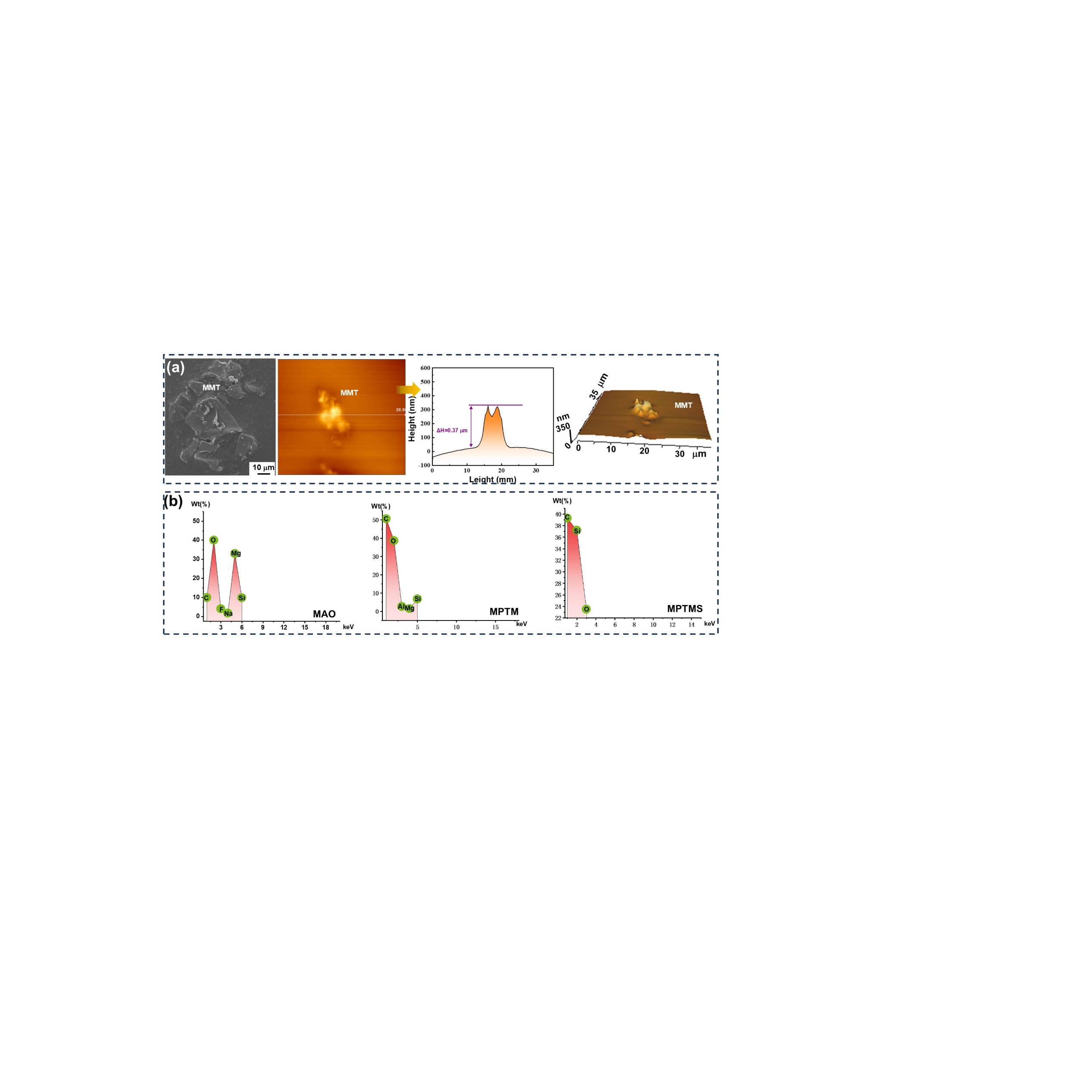


**Figure S15.** a) Enlarged SEM, AFM images and corresponding line cut of MPTM, b) EDS spectra indicating elemental composition of the samples.


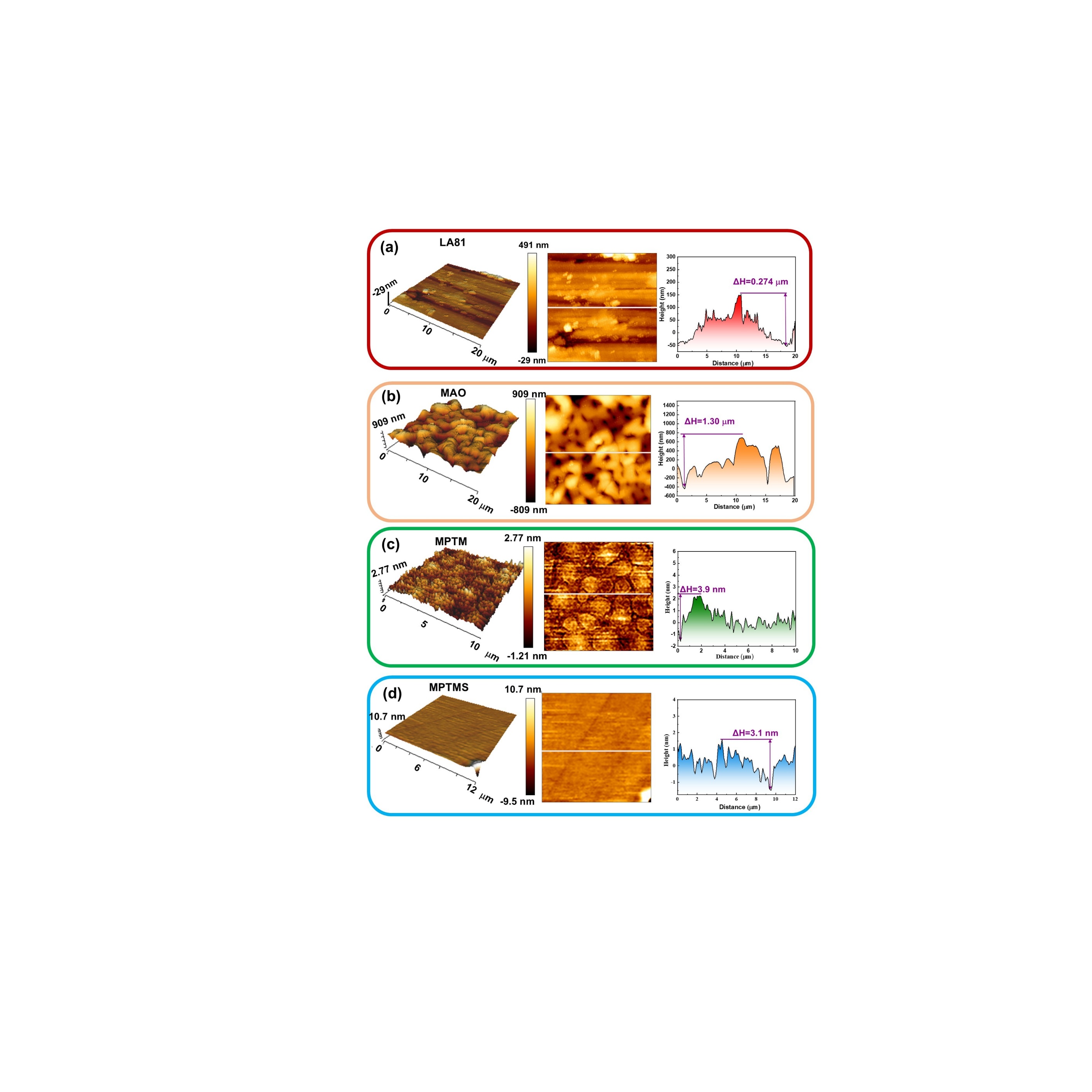


**Figure S16.** AFM images and corresponding surface height profiles of a) LA81, b) MAO, c) MPTM, and d) MPTMS coatings.


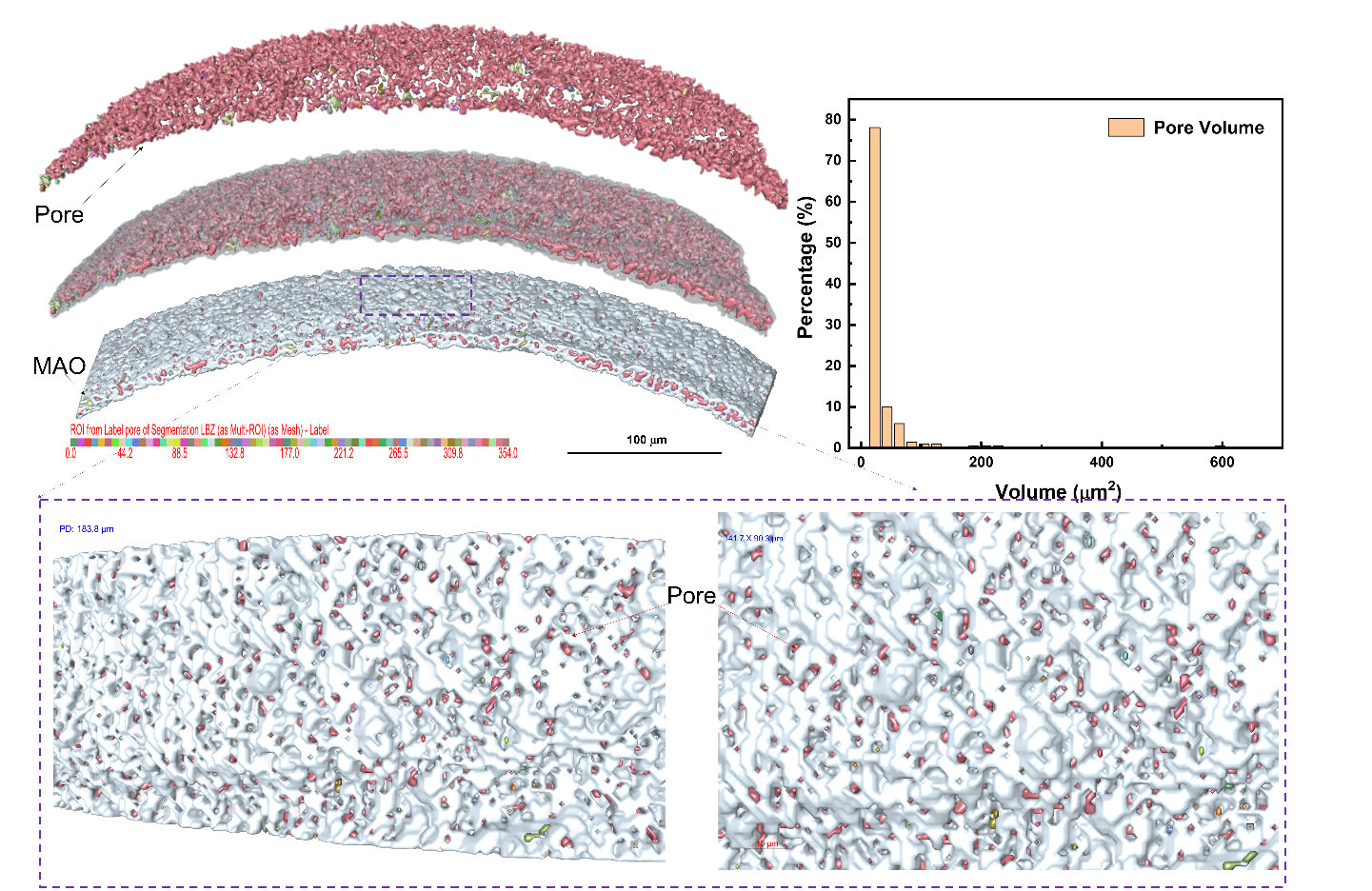


**Figure S17.** 3D topography and micropore volume distribution within MAO coating.


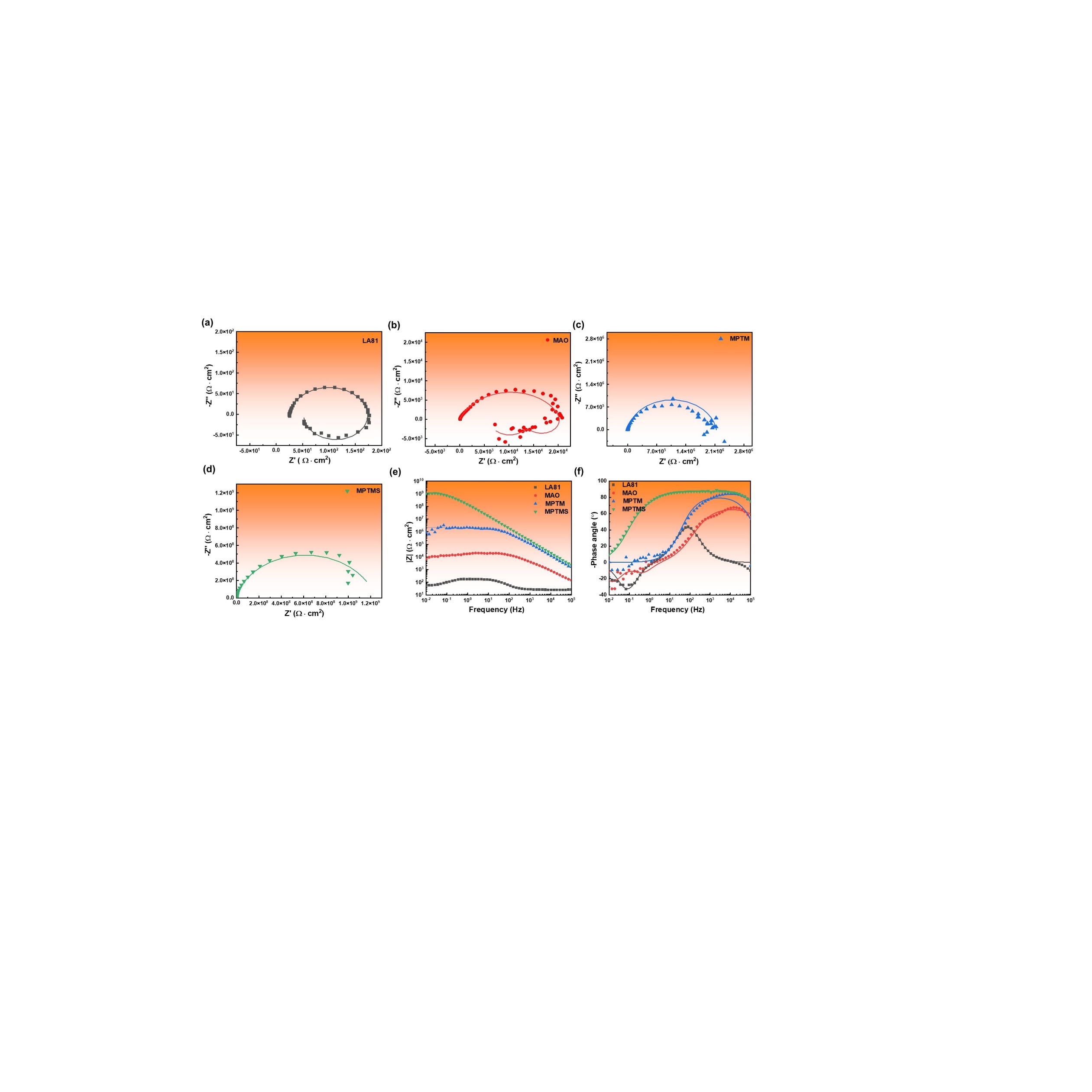


**Figure S18.** **(**a-d) Nyquist plots, and (e and f) Bode plots of different samples exposed to 3.5 wt% NaCl solution.


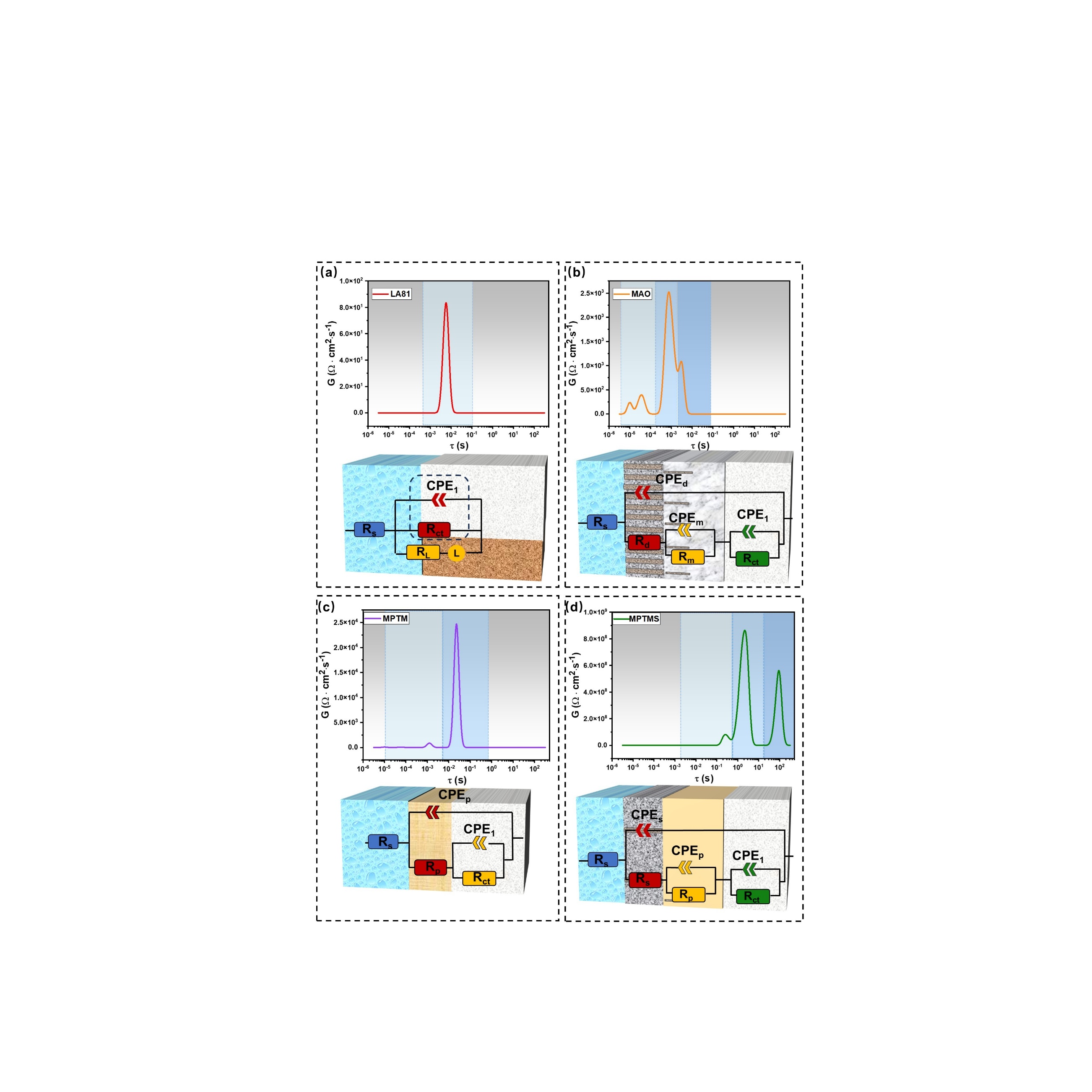


**Figure S19.** Distribution of relaxation time lengths in 3.5 wt% NaCl solution for a) LA81, b) MAO, c) MPTM and d) MPTMS samples and their corresponding equivalent circuit models.

**
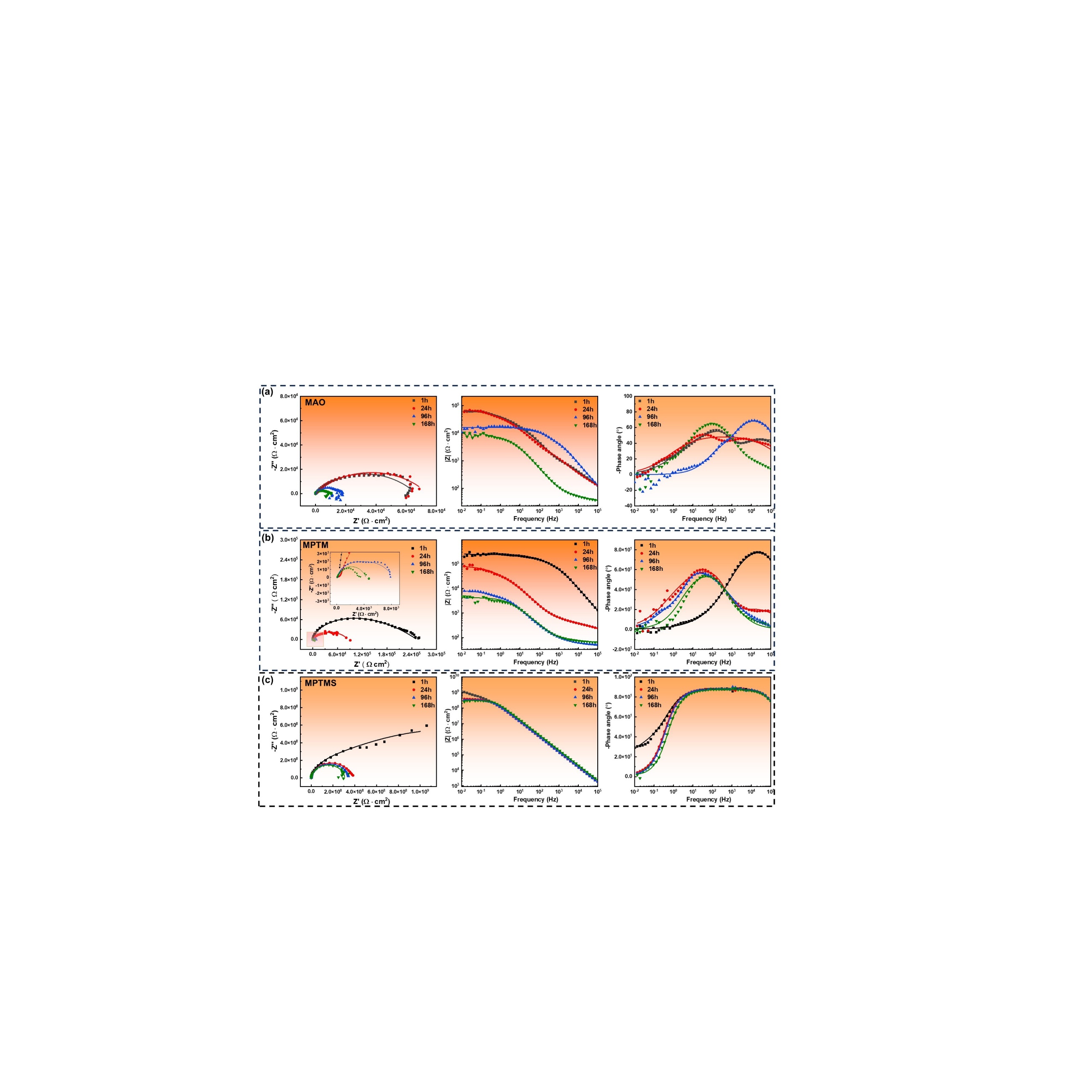
**

**Figure S20.** EIS spectra (Nyquist plots, Bode impedance modulus, and Bode phase angle) of a) MAO, b) MPTM, and c) MPTMS coatings in 3.5 wt% NaCl solution at different exposure time lengths.


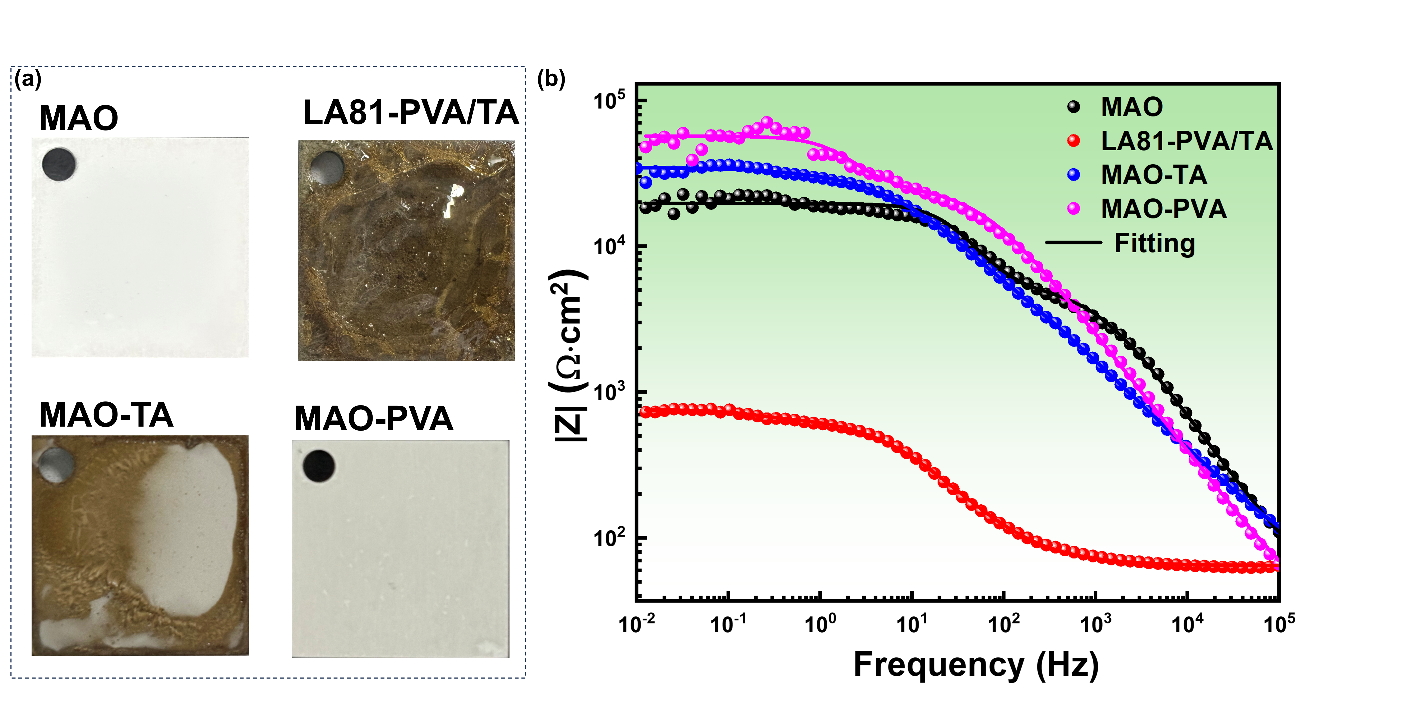


**Figure S21.** a) Optical image and b) EIS modulus plots of MAO, LA81-PVA-TA, MAO-TA, and MAO-PVA coatings.


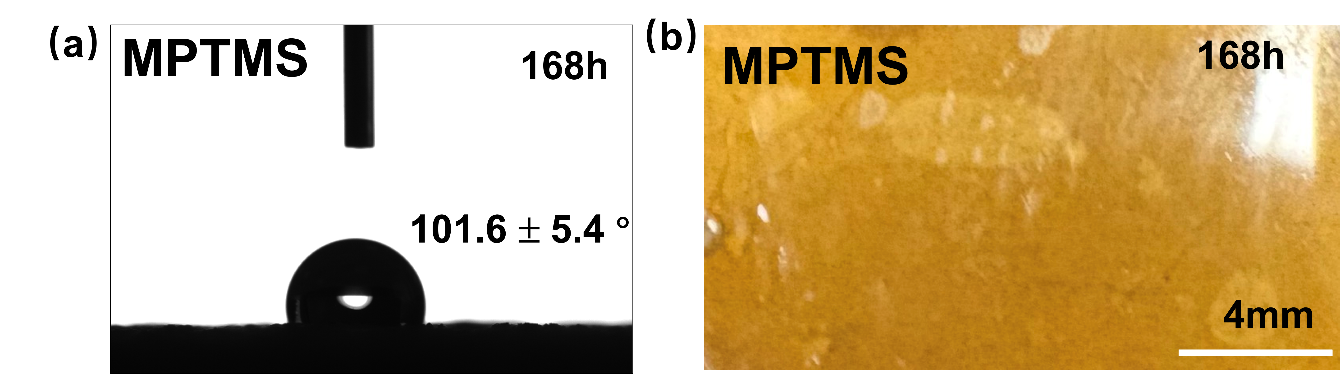


**Figure S22.** a) Static contact angle, and digital photo b) of MPTMS coating after soaking in 3.5% NaCl solution for 168 h.

**
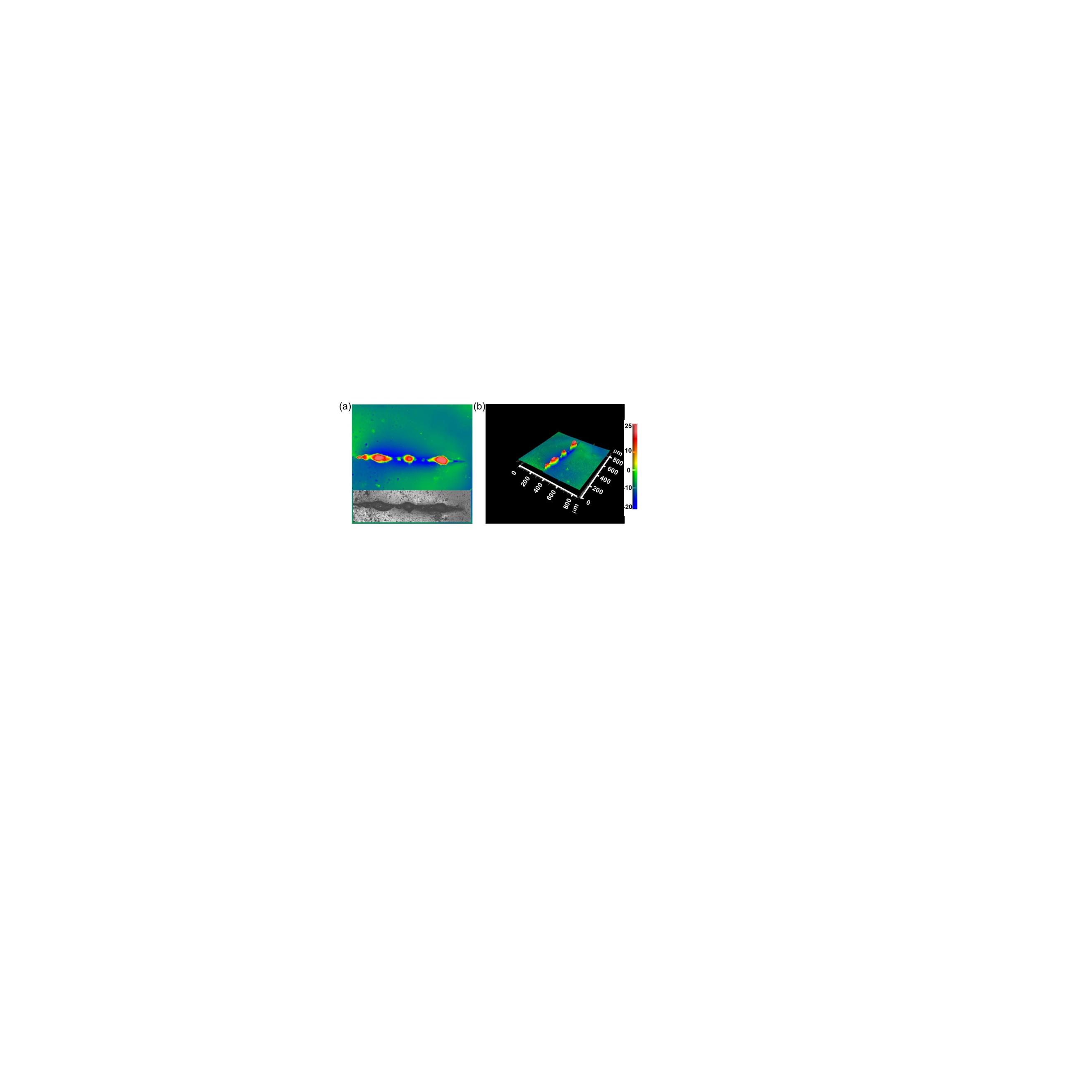
**

**Figure S23.** a) Laser confocal photograph and b) 3D macroscopic morphology of hydrophilic self-healing MPTM coating swelling upon water exposure.


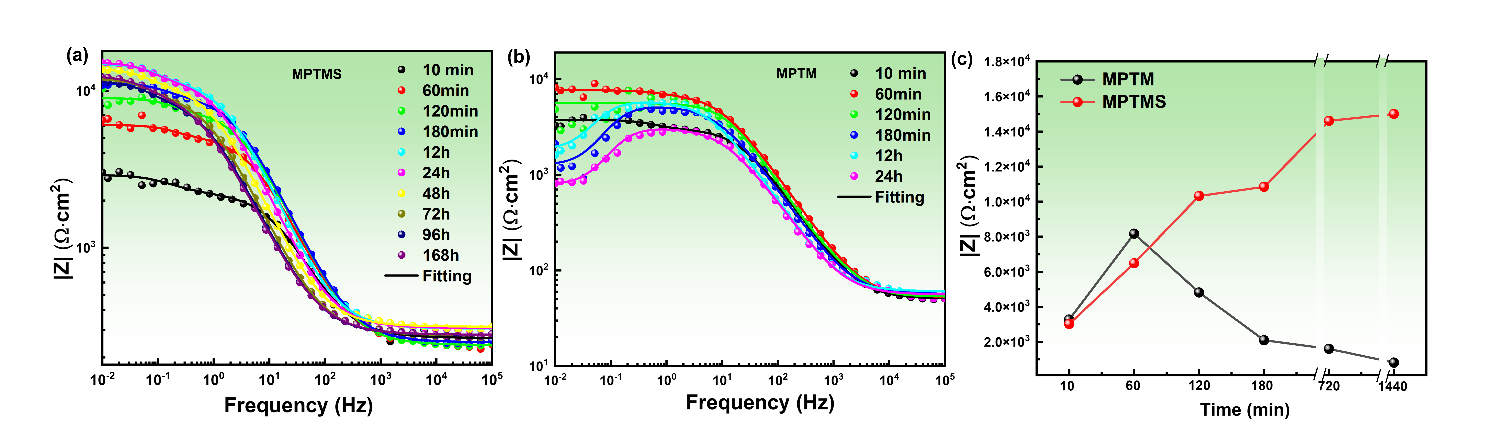


**Figure S24.** EIS of damaged coatings immersed in 3.5 wt% NaCl solution over extended periods: a) MPTMS-coated sample; b) MPTM-coated sample; c) evolution of the impedance modulus at 0.01 Hz during long-term immersion.

**
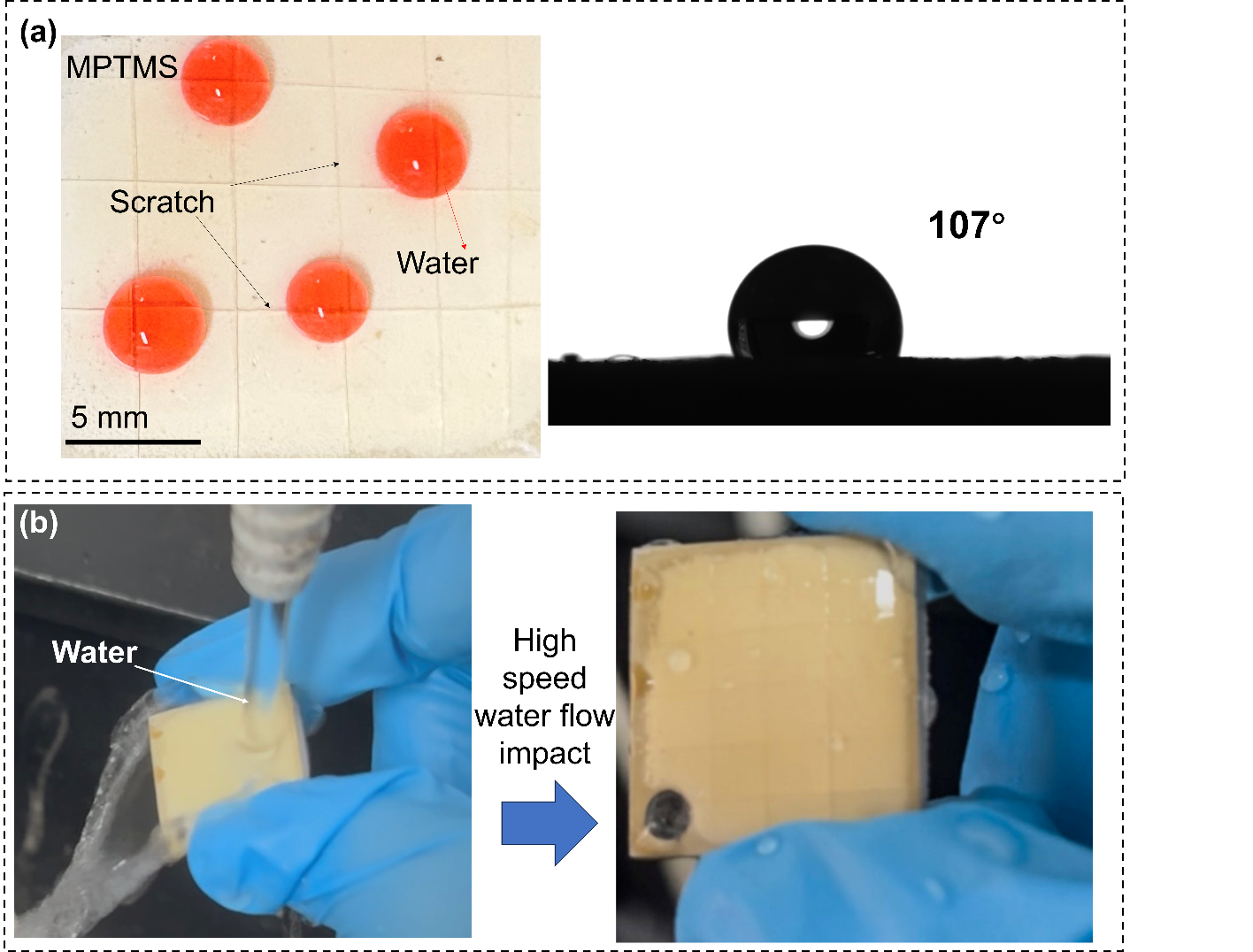
**

**Figure S25.** a) Macroscopic images showing the hydrophobic surface and static water contact angle of MPTMS coating after mechanical damage; b) macroscopic images of surface wettability of MPTMS coating after high-speed water flow impact following damage.


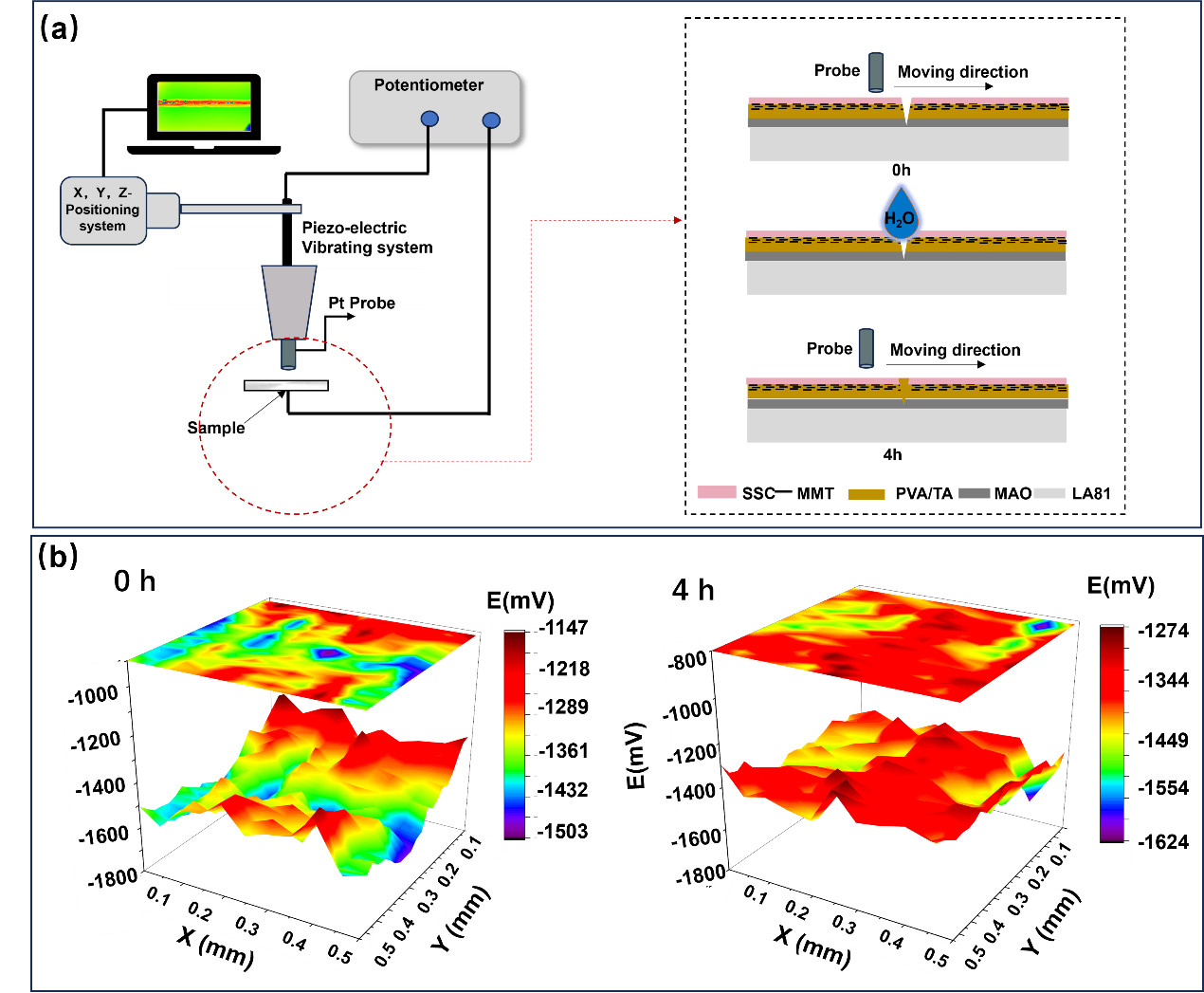


**Figure S26.** (a) Schematic of SKP testing and (b) 3D voltage potential maps obtained by SKP after scratching and during the self-healing process of MPTMS coating.


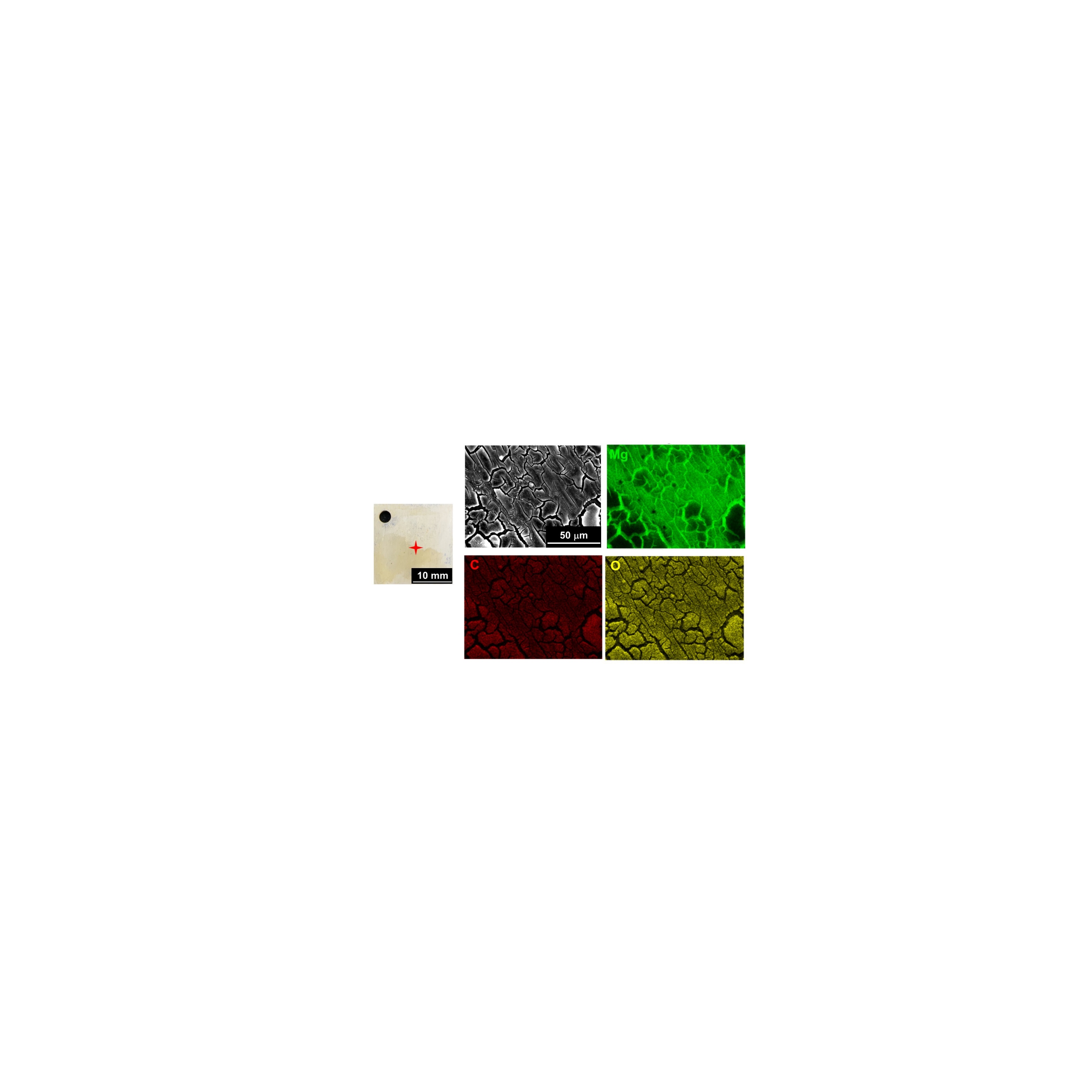


**Figure S27.** Digital photo, SEM micrograph and EDS mapping of Mg-Li alloy LA81 after immersion in 10 g/L tannic acid solution for 10 min.


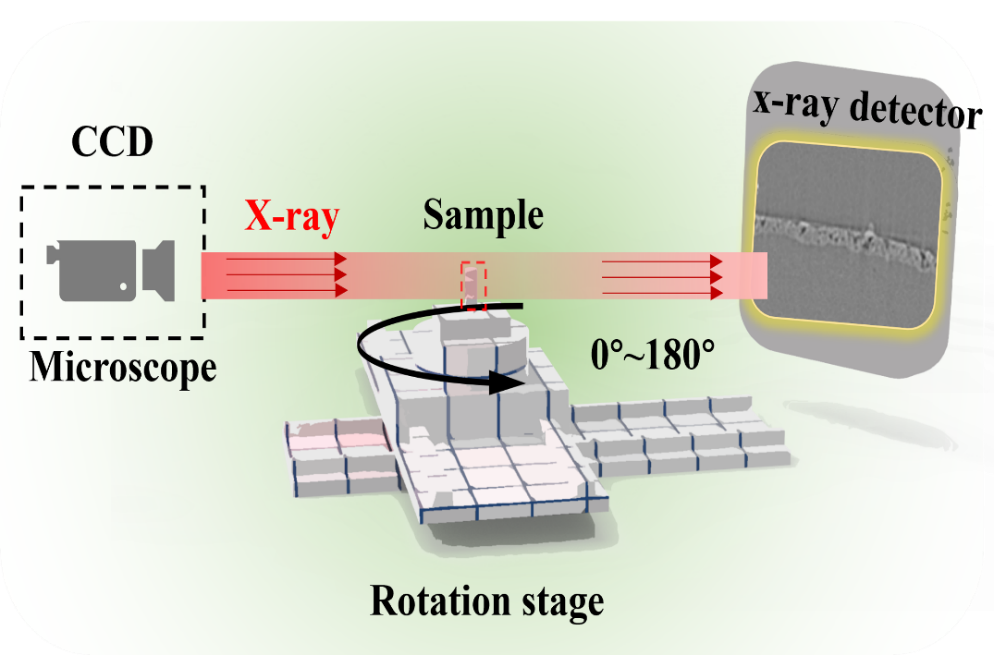


**Figure S28.** Schematic illustration of synchrotron X-ray computed tomography.
